# Competing adaptations maintain nonadaptive variation in a wild cricket population

**DOI:** 10.1073/pnas.2317879121

**Published:** 2024-08-01

**Authors:** Jack G. Rayner, Franca Eichenberger, Jessica V. A. Bainbridge, Shangzhe Zhang, Xiao Zhang, Leeban H. Yusuf, Susan Balenger, Oscar E. Gaggiotti, Nathan W. Bailey

**Affiliations:** ^a^Department of Biology, University of Maryland, College Park, MD 20740; ^b^Centre for Biological Diversity, University of St Andrews, St Andrews KY16 9TH, United Kingdom; ^c^Department of Ecology and Evolution, University of Lausanne, Lausanne 1015, Switzerland; ^d^Tianjin Key Laboratory of Conservation and Utilization of Animal Diversity, College of Life Sciences, Tianjin Normal University, Tianjin 300387, China; ^e^College of Biological Sciences, University of Minnesota, St. Paul, MN 55108

**Keywords:** adaptation, polymorphism, wild populations, *Teleogryllus oceanicus*, epistasis

## Abstract

How emerging adaptive variants interact is an important factor in the evolution of wild populations, but the opportunity to empirically study this interaction is rare. We recently documented the emergence of an adaptive phenotype “curly-wing” in Hawaiian populations of field crickets (*Teleogryllus oceanicus*). Curly-wing inhibits males’ ability to sing, protecting them from eavesdropping parasitoid flies (*Ormia ochracea*). Surprisingly, curly-wing co-occurs with similarly protective silent “flatwing” phenotypes in multiple populations, in which neither phenotype has spread to fixation. These two phenotypes are frequently coexpressed, but since either sufficiently reduces song amplitude to evade the fly, their coexpression confers no additional fitness benefit. Numerous “off-target” phenotypic changes are known to accompany flatwing, and we find that curly-wing, too, negatively impacts male courtship ability and affects mass and survival of females under lab conditions. We show through crosses and genomic and mRNA sequencing that curly-wing expression is associated with variation on a single autosome. In parallel analyses of flatwing, our results reinforce previous findings of X-linked single-locus inheritance. By combining insights into the genetic architecture of these alternative phenotypes with simulations and field observations, we show that the co-occurrence of these two adaptations impedes either from fixing, despite extreme fitness benefits, due to fitness epistasis. This co-occurrence of similar adaptive forms in the same populations might be more common than is generally considered and could be an important force inhibiting adaptive evolution in wild populations of sexually reproducing organisms.

## Significance

Understanding the ability of wild populations to adapt under extreme selection is a pressing challenge in contemporary biology. In our study of wild cricket populations evolving under extreme selection against male song, we find that two adaptive reduced-song phenotypes have repeatedly emerged in the same populations, demonstrating a remarkably rapid and effective adaptive response. However, because these phenotypes are associated with different genomic regions and there is no benefit to expressing both as opposed to just one, their co-occurrence actually impedes the spread of either mutation. The co-occurrence of adaptations with similar functions may be more common than is generally appreciated, and we find that this can considerably reduce the ability of wild populations to respond to selection.

Mutations that confer strong fitness benefits are expected to spread through populations. However, adaptive mutations do not arise in isolation. They interact at the organismal level with other fitness-associated alleles in the genome (1) and at the population level with other segregating alleles (2). Within a population, multiple beneficial mutations might emerge and segregate contemporaneously. In this case, an adaptive mutation’s spread also depends on its likelihood of recombining into the same genome as other adaptive mutations (3, 4). In asexual species, recombination is unlikely, leading alternative adaptations to compete for fixation [e.g., clonal interference (5)]. In sexual species, selective interference between adaptive mutations is thought to be less important as mutations can recombine into the same genome, though it can still occur, particularly when recombination is suppressed. Models examining these scenarios frequently assume that individuals carrying multiple adaptive mutations will have greater fitness than individuals carrying just one (6, 7). However, it is widely appreciated that this is not necessarily true, for example, if the loci in question interact epistatically (8). Similar adaptations frequently emerge in lineages evolving under similar selection pressures (9, 10) and can arise through independent genetic changes (11–14). In the scenario where similar adaptations co-occur in the same population, fitness epistasis—i.e., nonadditive effects on fitness—might arise if their coexpression does not confer additional fitness advantage relative to individuals expressing one or the other.

Here, we investigate a scenario of co-occurring adaptations in a system where multiple phenotypes—similarly adaptive under the same selection pressure, but through diverse morphological changes—have recently emerged across populations of the field cricket *Teleogryllus oceanicus*. Male crickets ordinarily produce song to attract females by rubbing their two forewings together, causing scraper and file structures on opposite wings to make contact and produce sound (15). However, Hawaiian populations of *T. oceanicus* are attacked by an introduced endoparasitoid fly, *Ormia ochracea*, which uses cricket song to locate hosts for its larvae (16). The fly imposes extremely strong selection against male cricket song, under which researchers have observed the repeated emergence and spread of different song-reducing phenotypes in Hawaiian populations. First, Zuk et al. (17) observed the emergence of “flatwing” phenotypes, which remove male ability to sing via loss or reduction of sound producing structures ordinarily present on the male wing. Flatwing variants have spread through populations on at least three different islands: Kauai, Oahu, and Hawaii, since they were observed in 2003. Of the three flatwing phenotypes from across these islands that have been subject to genetic analysis, all are underpinned by X-linked mutations (13, 18–20), but show differing patterns of genomic association that suggest the phenotypes arose independently (13, 18).

**Fig. 1.**
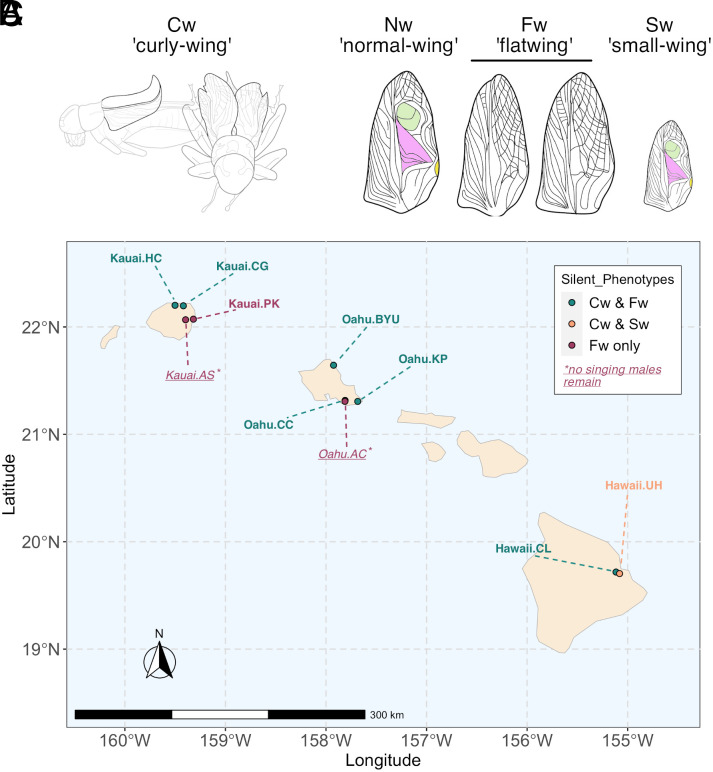
Presence of male-silencing *T. oceanicus* phenotypes across Hawaii. (*A*) Side- and front-view diagrams of Cw morphology, showing unusually curled forewings which would ordinarily sit flat. (*B*) Diagrams of male forewings illustrating singing-capable, normal-wing (Nw) venation, alongside that of two song-reducing phenotypes: flatwing (Fw) and small-wing (Sw). The two Fw diagrams illustrate variation in Fw morphology between islands and populations in the degree of reduction of principal sound-producing structures (highlighted in color in the diagrams of normal-wing venation). (*C*) Distribution of reduced-song phenotypes across the Hawaiian archipelago. Text labels refer to study populations, with underlined italics indicating that no singing males remain in Kauai.AS and Oahu.AC populations. Colors indicate which silencing phenotypes are present in each population.

We recently documented the emergence of two more reduced-song wing phenotypes, “curly-wing” and “small-wing”, in Hawaiian *T. oceanicus* populations (21) (Fig. 1 *A* and *B*) (22): See also two other recently described song-reducing phenotypes (22, 23). Like flatwing (Fig. 1*B*), these phenotypes benefit males by reducing or eliminating their ability to sing, allowing them to evade detection by *O. ochracea* (21, 24). Curly-wing and small-wing can be visibly expressed by females, whereas the flatwing mutation does not affect female wings. Usefully, flatwing, curly-wing, and small-wing phenotypes are readily distinguishable, allowing us to document their contemporaneous spread. While we have only observed small-wing in two nearby populations, curly-wing—like flatwing—is observed in several populations across the Hawaiian archipelago (13). We find that curly-wing and flatwing phenotypes are frequently present in the same populations, and are frequently coexpressed by males. Of the seven study populations in which we observe curly-wing phenotypes, flatwing phenotypes are also present in six, while in the seventh, curly-wing, small-wing, and rattling phenotypes all co-occur (25) (Fig. 1*C*). Because expression of either the curly-wing or flatwing phenotype is sufficient to protect males against parasitism (17, 21, 25) we hypothesize their co-occurrence might impede either’s fixation. This is because each nonadaptive, song-associated phenotype (i.e., wild-type 3D wing morphology, or normal-wing venation) is shielded from negative selection when coexpressed with the alternative song-reducing adaptation (i.e., flatwing venation, or curly-wing morphology). Consistent with this, song-associated phenotypes remain in all populations where two or more reduced-song morphs co-occur, in contrast with two populations in which flatwing alone spread to fixation such that no singing-capable males remain (Fig. 1*C*).

To test this, we investigated the genetic architecture and evolutionary dynamics of these cosegregating adaptive variants. Our first goal was to assess the heritability of curly-wing and identify associated genomic regions. Our second goal was to compare genetic and transcriptomic features of co-occurring curly-wing and flatwing phenotypes. Adaptive mutations often have negative fitness consequences for a range of related and unrelated traits (2, 26), so our third goal was to test consequences of curly-wing and flatwing expression in the context of male sexual advertisement, and on adult size and longevity in both sexes. Finally, we present simulations informed by our findings to evaluate the inference that co-occurrence of alternative adaptative phenotypes has impeded their adaptive spread in wild *T. oceanicus* populations, and describe conditions favoring such an outcome.

## Results

### Forewing Nomenclature.

All wing phenotypes studied here are expressed in forewings (i.e., tegmina) which crickets use to sing. Nomenclature follows Bailey et al. At the level of forewing venation, normal-wing (Nw) and flatwing (Fw) morphology correspond to the original descriptions by Zuk et al. At the level of 3-dimensional wing morphology, we refer to curly-wing (Cw) and wild-type (Wt) phenotypes, where Wt indicates the absence of Cw morphology (cf. Fig. 1*A*). By contrast with Cw, Wt forewings lie flush, right overlapping left, on the dorsal surface of the abdomen when at rest. Flatwing and curly-wing are therefore not opposite, but instead describe different male-silencing phenotypic adaptations which can be coexpressed (i.e., CwFw).

### Cw Is Highly Heritable and More Strongly Expressed by Females.

**Fig. 2.**
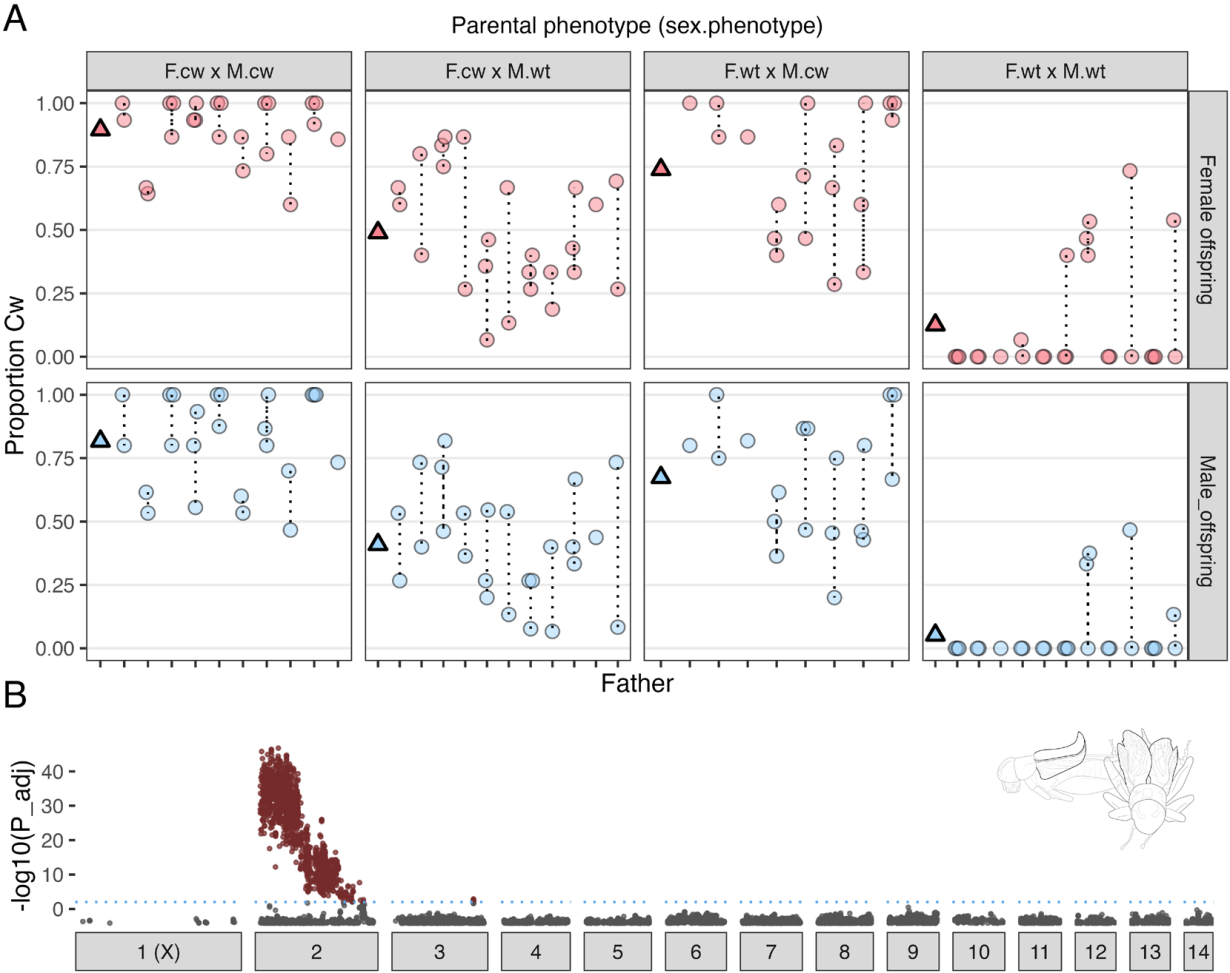
Inheritance and genetic architecture of Cw. (*A*) Each circle shows the proportion of full-sib F_1_ offspring expressing curly-wing following mating of the respective F_0_ male (X-axis) with a single female. Dotted lines connect half-sib families. Panels are separated into groups based on parental curly-wing phenotype (F.cw × M.cw = curly-wing dam mated with curly-wing sire, and so on), and by offspring sex. Triangles show mean values for each panel. (*B*) Genome-wide association significance (−log_10_ Bonferroni-adjusted P) between allelic variants and Cw phenotype. The dashed line indicates a *P*-value threshold of 0.01, and chromosomes are numbered along the X axis.

Half-sibling crosses, performed using laboratory stock originally derived from the Oahu.CC population (Fig. 1*C*), showed that Cw is heritable and segregates in a manner consistent with autosomal inheritance (Fig. 2*A*). In a linear mixed model, a random effect corresponding to parental identities explained 90% of variance in the proportion of Cw offspring. Replacing our response variable with average offspring curliness score (*SI Appendix*, Fig. S1 and Table S1) reduced the estimated R^2^ for the random effect term from 0.90 to 0.77, so we treat Cw as a discrete trait. Patterns of Fw inheritance were consistent with X-linkage previously observed (18, 19) (*SI Appendix*, Fig. S2). Among male offspring, there was no association between expression of Cw and Fw phenotypes, indicating that causal regions are located on different chromosomes (*SI Appendix*, Table S2): likely a key factor in their evolutionary interaction.

As expected in the case of Cw being caused by one or few colocalized major effect loci, Cw × Cw and Wt × Wt crosses produced predominantly Cw and Wt offspring, respectively (Fig. 2*A*), albeit with exceptions attributable to variable penetrance (i.e., incomplete penetrance of the Cw genotype, or expression of the Cw phenotype in heterozygous individuals). In crosses where just one parent visibly expressed Cw, paternal phenotype was more strongly associated with the proportion of Cw offspring (FatherCw × MotherCw interaction: X^2^_1_ = 6.613, *P* = 0.010; Fig. 2*A*). We also observed that Cw expression was affected by sex (X^2^_1_ = 24.816, *P* < 0.001), being less frequently expressed by males. A plausible explanation for the parent-of-origin effect is that Cw-expressing males were more likely to carry multiple copies of the underlying allele, relative to females who are more likely to express it in heterozygous state, and thus males were more likely to transmit it to offspring. Curliness was also positively associated with rearing density, albeit not significantly (X^2^_1_ = 3.284, P = 0.070; full model est. R^2^ of 0.922). Sex and rearing density influence expression, in the same direction, of an analogous curled wing phenotype, “curly”, in *Drosophila* (27, 28). In field crickets, sex and rearing density each affect development rate (29, 30), suggesting wing curliness might be influenced by growth rate, as in *Drosophila* (31).

### Cw is Genetically Associated with a Single Autosome, Chromosome 2.

We performed genome-wide association tests using RAD-seq data from 178 Cw and 197 Wt individuals from a single inbred F_2_ family derived from the Oahu.CC population, with 13,832 filtered RAD markers mapped to the *T. oceanicus* reference genome v.2.0 (32). We detected Cw-associated variants across nearly the full length of the 245 Mb Chr2 autosome, with the association strongest across the first ca. 85 Mb (Fig. 2*B*).

Chr2 was also highlighted for involvement in the Cw phenotype by differential expression analysis (*SI Appendix*, Fig. S3). One hundred genes were differentially expressed (DE) at P_adj_ < 0.05 between developing wings of Cw vs Wt males (DE_Cw_), whereas 179 were DE between those of Nw and Fw males (DE_Fw_). In both cases, DE genes were concentrated on chromosomes harboring the respective causative variants. Among DE_Cw_ genes, 54 (54%) were located on Chr2 (*SI Appendix*, Fig. S3). This represents strong overrepresentation given that Chr2 accounted for 12.56% of the 11,580 genes present in the RNAseq analysis which could be placed on one of the 14 chromosomes (X^2^_1_ = 96.000, *P* < 0.001). Moreover, 45 of these 54 genes were within the 0:85 Mb region strongly associated with Cw in the RAD-seq data. Of the 39 DE_Cw_ genes annotated by homology (blastx, e-value < 1e−6) with *Drosophila* proteins, four are involved in serine-type endopeptidase inhibitor activity; these “serpin” genes are associated with Cw-like phenotypes in *Drosophila* (33, 34). For example, RNAi-mediated knockdown of the serpin gene *Spn5* causes defective wing unfolding during *Drosophila* eclosion (34). In *T. oceanicus,* serpin genes are highly represented on Chr2, which harbors 13 of the 58 (22.5%) serpin genes found within the genome, whereas Chr2 accounts for 13.3% of all annotated genes (X^2^_1_ = 3.443, *P* = 0.064). Among other genes of interest, a homolog of dumpy, which in *Drosophila* is involved in apposition of dorsal and ventral imaginal disc-derived wing surfaces (35), showed evidence of upregulation in Cw samples, though not significantly so (*P*_adj._ = 0.078).

Among the 179 DE_Fw_ genes, 46 (25.70%) were located on the X chromosome (cf. 12.0% of genes in the filtered transcriptome; X^2^_1_ = 30.264, *P* < 0.001) (*SI Appendix*, Fig. S3). We found that a predicted, unannotated gene 7 kb downstream of the flatwing-associated annotated gene doublesex (dsx), was down-regulated in Fw samples, was among the most strongly DE genes (ranked 8th; P_adj._ = 1.2e−14). Downregulation of *dsx* has previously been observed in developing Fw wing tissue, at the instar prior to that sampled in the current study (13).

### Evidence Supporting a Large Cw-Associated Inversion.

The large Cw-associated region on Chr2, without an obvious peak of genetic association (Fig. 2*B*), could implicate a large structural variant such as a chromosomal inversion. Using existing whole genome sequencing (WGS) data collected in 2017 from the wild Oahu.CC individuals from which our lab stock was derived, we found strong evidence of an inversion in this region. Specifically, principal component analysis (PCA) separated samples into three clusters representing homozygotes for the two divergent nonrecombining haplotypes with an intermediate cluster of heterozygous samples (36) (*SI Appendix*, Figs. S5, S6). Samples from these clusters showed drastically different rates of heterozygosity and strong linkage within the first 80 Mb of Chr2, consistent with expectations of a segregating inversion (37, 38) (*SI Appendix*, Fig. S6). We used *Delly2* to predict breakpoints associated with large (>5 Mb) inversions in this region based on paired-end read alignments (39). After filtering for location and expected frequencies across samples based on PCA clustering, there remained a large predicted inversion corresponding to our observations between 7.5 and 80 Mb on Chr2. This approach has a high false positive rate with short read sequencing data, but for convenience, we henceforth treat the inversion as spanning the region of 7.5 to 80 Mb on Chr2.

### Genotype–Phenotype Associations Across Wild Populations.

Cw and Fw phenotypes co-occur—i.e., are found together in the same populations—with surprising frequency. To investigate the genomic architecture of Cw and Fw phenotypes in wild populations, we analyzed WGS data from samples collected in 2021 and 2022 from males of known phenotype from the focal Oahu.CC population (13 Cw, 17 Wt; 8 Nw, 22 Fw), and from populations on two other Hawaiian islands: Kauai.CG (19 Cw, 11 Wt; 10 Fw, 20 Fw), and Hawaii.UH (18 Wt, 12 Cw, all Nw) (Fig. 1). Neither Cw nor Fw phenotypes have reached fixation in any of these populations, and seven males from Oahu.CC and 12 males from Kauai.CG coexpressed Cw and Fw—i.e., the two phenotypes were expressed together in the same individual. Given prior knowledge of their genetic architectures, we focused analyses of Cw and Fw phenotypes on Chr2 and the X, respectively. For analyses of Fw, Oahu.CC data were combined with data collected from the same population in 2017 for a full sample of 50 individuals (18 Nw, 32 Fw).

**Fig. 3.**
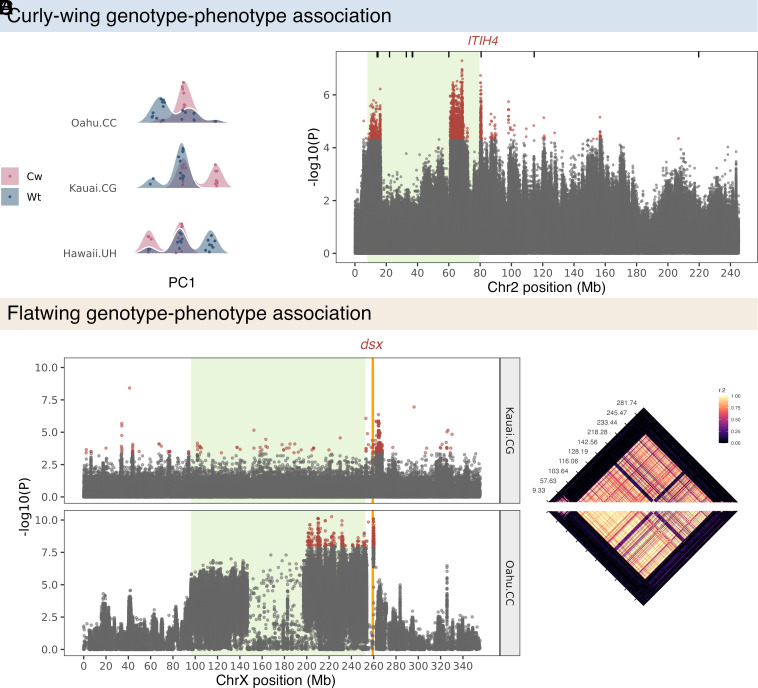
Genomic regions associated with Cw and Fw. (*A*) PC1 clustering across populations using SNPs within 7.5:80 Mb region of Chr2. Points are jittered on the Y-axis to fill the density distribution illustrated by curves, and both are colored by sample phenotype. (*B*) SNP-wise association tests between Cw and Wt phenotypes across all populations. Vertical lines on top show locations of genes with an annotated function in serine-type endopeptidase inhibitor activity. (*C*) SNP-wise association tests between Fw and Nw phenotypes within Kauai.CG and Oahu.CC populations, on the X chromosome. The vertical orange line indicates the position of *dsx*. (*D*) Linkage across the full X chromosome in Oahu.CC (*Top*) and Kauai.CG (*Bottom*). In panels *B* and *C*, green filled areas highlight regions of putative inversions, and red points are 99.9% significance outliers.

We observed strong association between Cw and genetic variation across the region of the predicted inversion at 7.5:80 Mb on Chr2. The pattern of sample clustering across populations was again consistent with the presence of an inversion in this region, separating samples from all populations into three discrete clusters on PC1 (explaining 46% of variance) (Fig. 3*A*). The inferred frequency of haplotypes in this region differed between Cw and Wt samples in all three populations (Wilcoxon rank-sum test: P_Hawaii.UH_ = 0.005; P_Kauai.CG_ = 0.005; P_Oahu.CC_ = 0.011). However, the pattern of Cw–PC1 association in Hawaii.UH was opposite that of Kauai.CG and Oahu.CC (Fig. 3*A*). To investigate this incongruity, we performed an F_ST_ scan [10 kb window, 10 kb step size, using Weir and Cockham’s F_ST_ implemented in vcftools (40)] between samples inferred to be homozygous for inverted haplotypes in Kauai.CG and Hawaii.UH, but which expressed opposite phenotypes. This analysis highlighted three regions of striking divergence: ca. 7.5:15 Mb, 60:70 Mb, and 80 Mb (*SI Appendix*, Fig. S7). Visualization of linkage and heterozygosity along Chr2 also revealed distinct patterns in Hawaii.UH between 7.5:80 Mb compared with Oahu.CC and Kauai.CG (*SI Appendix*, Fig. S8), suggesting recurrent chromosomal rearrangements in this region (38, 41, 42). We anticipated the Cw-associated variant(s) localizes to one of these three high F_ST_ windows, as variants in these regions will be statistically associated with the inversion in each population via linkage, but could show opposite patterns of association between populations (i.e., gametic coupling in Kauai.CC and Oahu.CC, but repulsion in Hawaii.UH).

In an association test between Cw and Chr2 SNPs across samples from all three populations (N = 2,245,749 SNPs), 3,378 SNPs were significantly (P_adj_ < 0.05) associated with Cw. Outlier SNPs were concentrated within or immediately adjacent to the putative inversion (Fig. 3*B*), consistent with the results from our mapping family (Fig. 2*B*), and within candidate regions highlighted by the F_ST_ analysis above. While these SNPs showed clear divergence between Cw and Wt phenotypes (*SI Appendix*, Fig. S7), there were also exceptions; in particular, four of the 46 Wt samples were consistently genotyped for Cw-associated variants (*SI Appendix*, Fig. S9), which we expect is due to incomplete penetrance of the Cw mutation(s) (Fig. 2*A*). One of these four samples was a small-wing male (Fig. 1*A*) in which the Cw phenotype may be less easily distinguished. Of the top 100 associated SNPs, four were located within annotated genes: *ADAM10*, *CASC1, IDHP, and ITIH4*. The SNP within the annotated region of *ITIH4* (at 80,625,117 bp) is near the putative inversion breakpoint (*SI Appendix*, Fig. S6). *ITIH4* has an annotated function in serine-type endopeptidase inhibitor activity [GO:0004867; implicated in Cw-like phenotypes in *Drosophila* (33, 34)], and was up-regulated in developing wing tissue of Cw males (P_unadj._ = 0.014). There was also a high density of DE_Cw_ genes in this region (*SI Appendix*, Fig. S10).

Flatwing phenotypes have been repeatedly associated with *dsx* at genomic and transcriptomic levels (13). We investigated Fw-associated variants separately in Oahu.CC and Kauai.CG (N = 897,136 and 377,327 X-linked SNPs, respectively) as prior analyses have shown that Fw variants from Oahu and Kauai have independent genetic architectures (Fig. 3*C*) (13, 18). In Kauai.CG, a cluster of 99.9% significance outlier SNPs was localized at 262 to 264 Mb (Padj. = 0.057), and two singleton SNPs at 41.28 and 296.16 Mb reached significance (Padj. < 0.05). In Oahu.CC, Fw-associated SNPs were found across nearly the full range of X chromosome and a prominent cluster of 99.9% significance outliers surrounded *dsx* at ca. 259 to 260 Mb (Fig. 3*C*). Twenty-four of the top 50 SNPs in the Oahu.CC population were within 1 Mb of *dsx* (259.05 to 259.44 Mb), whereas in Kauai.CG, 31 were located nearby, between 262 to 265 Mb. These SNPs showed consistent and strong, but imperfect genotypic divergence between Nw and Fw samples in each population (*SI Appendix*, Fig. S11). In both populations, we observed very strong linkage between SNPs across a very large portion of the X chromosome (Fig. 3*D*) and following PCA samples grouped into two discrete clusters (*SI Appendix*, Fig. S12), supporting the presence of another very large inversion which we suspect contributed to the low density of RAD SNPs on the X in our inbred mapping family (Fig. 2*B*). Inspection of linkage patterns in both populations suggested this putative inversion spans ca. 95.7 to 253.2 Mb. Despite the proximity of this inversion to Fw-associated SNP outlier clusters in each population, Fw and Nw samples were not separated on PC1 or PC2 in either population (*SI Appendix*, Fig. S9). This indicates that the inversion is not causally associated with Fw, though there was a statistical association between the inversion and Fw in Oahu.CC (Wilcoxon rank-sum test: *P* = 0.001), reflected in the signal of Fw-association across the region (Fig. 3*C*). It appears likely that a degree of hitchhiking between the inversion and the physically proximate Fw-associated locus at 259 Mb accounts for this pattern in Oahu.CC (13, 18).

### Correlated Phenotypic Consequences.

While Cw and Fw do not appear to confer greater fitness benefits in the coexpressed state, mutations that spread under strong selection frequently also incur fitness costs, e.g., via pleiotropic effects and genetic hitchhiking. Such negative fitness consequences are more likely in cases where large effect mutations are favored due to extreme displacement of a population from a fitness optimum (43), as in *T. oceanicus* populations parasitized by *O. ochracea* (20, 44), and would likely combine additively. Thus, males expressing both phenotypes could actually be disadvantaged, and female carriers might also suffer negative fitness-associated consequences of Cw and Fw, despite male-limited fitness benefits (females being obligately silent).

**Fig. 4.**
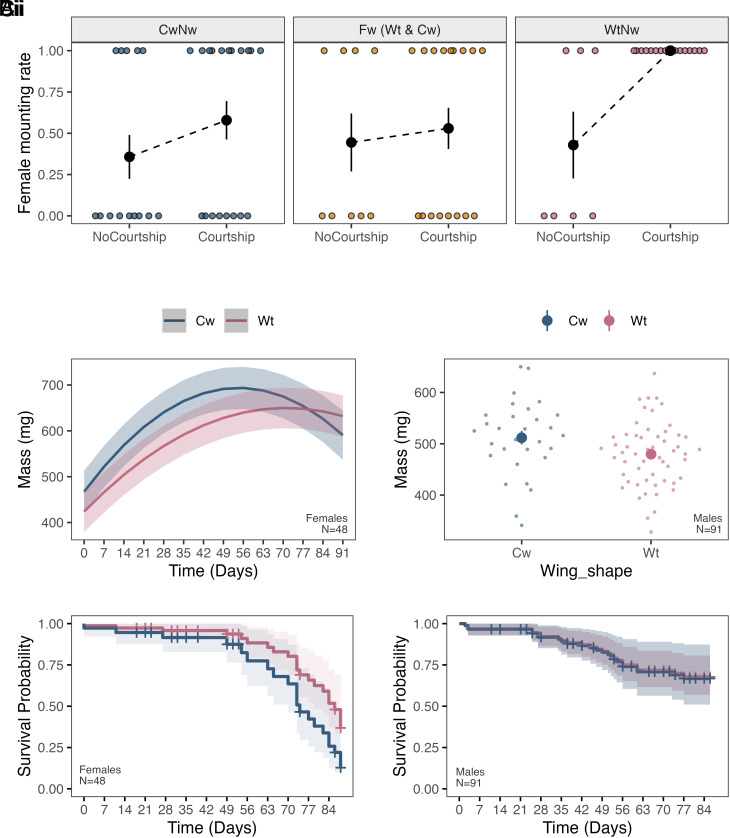
Correlated fitness consequences of the adaptive *Cw* mutation. (*A*) Effects of courtship (measured by attempt to produce courtship song) by males of different wing phenotypes upon rates of female mounting (i.e., decision to mate). Black points and error bars show means ± SE. (*B*) (i) Predicted mass ± SE across adult ages in females. (ii) Observed mass at 14 d posteclosion for males, with large solid points showing means. Vertical bars indicate ± SE but are partially obscured by mean symbols. (*C*) (i), Probability of survival (solid lines) at adulthood based on proportional hazards regression of Wt and Cw phenotypes in (i) females and (ii) males, with shading indicating 95% CI.

In grylline field cricket species such as *T. oceanicus*, females exert control over mating interactions as they must mount males in order for mating to occur. Male song is an important courtship trait, and males that can sing have a strong advantage in mating interactions (24). In mating trials, we found female mate acceptance differed predictably between song-producing normal-wing (WtNw), and flatwing (WtFw and CwFw) and Cw (CwNw) males in accordance with their ability to produce courtship song (binomial GLM of female decision to mount: MaleMorph × Courtship X^2^_2_ = 7.54, *P* = 0.023). Among males that attempted to produce courtship song by elevating their forewings and stridulating, Cw and Fw phenotypes were strongly disadvantaged compared with those expressing WtNw morphology; courtship by the latter consistently elicited much higher rates female mounting (Fig. 4*A*; *SI Appendix*, Fig. S13). While the fitness cost associated with reduced courtship ability is evidently substantial, the spread of songless phenotypes in *T. oceanicus* across the Hawaiian archipelago, in some cases to fixation (21, 45), implies that this cost is secondary to fitness benefits gained from evading detection by *O. ochracea* (24).

Apart from effects of reduced signaling ability on male fitness, adaptive mutations might exert other pleiotropic or indirect fitness consequences. Most strikingly, females expressing Cw morphology had significantly reduced longevity (Hazards ratio = 2.06; *P* < 0.001; *SI Appendix*, Table S3), suggesting negative fitness effects of the phenotype in females, who also do not benefit directly from loss of song (Fig. 4*Ci*). Neither Cw nor Fw phenotypes appeared to affect male longevity, though we recorded low male mortality in general over the course of the experiment (Fig. 4*Cii* and *SI Appendix*, Table S3). Similarly, male mass at 14 d adulthood was not associated with wing shape (Cw vs. Wt) or wing venation (Nw vs. Fw) (Fig. 4*Bii*) (*SI Appendix*, Table S4). However, Cw females typically had greater mass (Fig. 4*Bi*), but this effect diminished at older ages. This pattern was also reflected in scaled mass index, sometimes used as a measure of body condition (46). Fitness consequences of greater body mass in female carriers of Cw are thus unclear, and we also note that differences in survival were more pronounced at older ages, when fitness impacts may be less severe (Fig. 4*Ci* and *SI Appendix*, Tables S3–S5).

### Interacting Mutations Under Conditions of Additive and Nonadditive Benefits.

Informed by the nonoverlapping genetic architectures of the two phenotypes, we evaluated the general prediction that similar adaptive variants (such as Cw and Fw) segregating in the same population would impede either from reaching fixation, using Wright–Fisher simulations implemented in SLiM v4.0.1 (47). Consistent with this, we found that nonadditive fitness benefits of co-occurring adaptive mutations—i.e., adaptive mutations segregating in the same population—impede the fixation of either one under strong selection.

In a population of 500 sexually reproducing individuals, we introduced two unlinked mutations *m1* and *m2*, each, to a separate random subset of 5 male genomes. Simulations were run under four scenarios with 10,000 simulations each. In all cases, mutations *m1* (autosomal with dominance coefficients 0.5, 0.75, or 1) and *m2* (X-linked) each conferred a selective advantage of +0.3 in the absence of the alternative mutation, and fitness benefits were male-specific. In the ‘additive’ benefit scenario, individuals expressing *m1* and *m2* had relative fitness 1.6. In the ‘nonadditive’ benefit scenario, individuals expressing both *m1* and *m2* had relative fitness 1.3—the same as individuals carrying just one or the other. In a ‘negative’ scenario, individuals expressing both mutations had relative fitness of 1.2, simulating a scenario in which mutations confer nonadditive fitness benefits, but additive pleiotropic fitness costs. For comparison, we also simulated ‘single’ scenarios, in which either mutation alone arose.

**Fig. 5.**
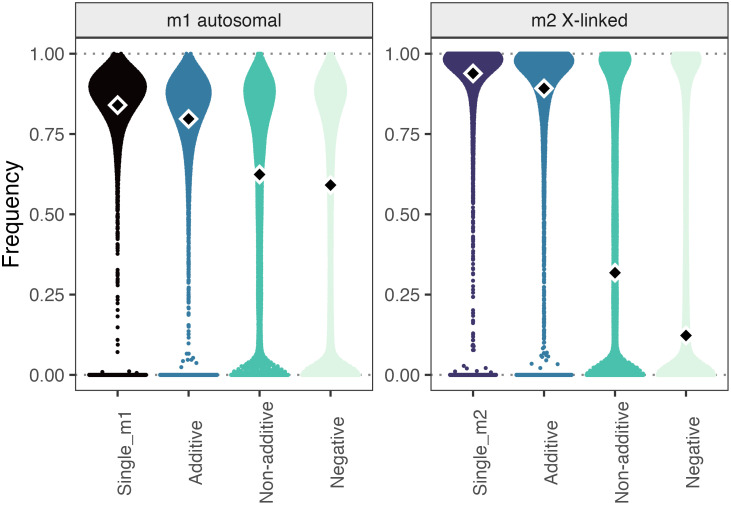
Effects of fitness epistasis on the spread of competing mutations. Frequencies after 100 generations of mutations *m1* (autosomal, dominance coefficient of 0.75) and *m2* (X-linked) under scenarios where the mutations combine to have additive, nonadditive, or negative effects on fitness of individuals expressing both, versus those expressing either one. Note that frequencies of co-occurring mutations are from the same simulations. Diamonds show median frequencies, and points are clustered along the X-axis according to their density distribution.

Mutation frequencies after 100 and 250 generations supported our inference that nonadditive benefits of Cw and Fw coexpression impede fixation of either variant. Each adaptive mutation spread rapidly if they independently emerged as ‘single’ mutations in the population. This resembles the observed emergence and spread of Fw to near-fixation (>95%) within 20 generations (17, 20)—and its subsequent fixation—in two *T. oceanicus* populations (21). Mutations also spread rapidly if they emerged contemporaneously and conferred additive fitness benefits when both expressed by the same individual (Fig. 5).

However, the spread of both mutations was impeded relative to either of the above scenarios if they conferred nonadditive benefits in the combined state—particularly when negative pleiotropic effects combined additively (the ‘negative’ scenario) (Fig. 5). These latter two situations appear most applicable to the Cw/Fw interaction, where coexpression confers no additional benefit and may reduce fitness through additive, negative pleiotropic effects. For reference, Cw and Fw have each been present in the Oahu.CC population, in which Cw was first identified, since at least 2012 (ca. >40 generations at the time of publication). In 2022, Cw and Fw phenotypes were expressed by 50% and 78% of 48 sampled males in this population, respectively, which appears broadly consistent with simulation results under nonadditive fitness benefits. The general pattern of nonadditive fitness benefits impeding spread of one or both mutations was consistent across dominance coefficients of 0.5, 0.75, and 1 for *m1*, after 250 generations, and in the scenario where both mutations were autosomal (*SI Appendix*, Figs. S14–S19).

## Discussion

Evolutionary dynamics following the contemporaneous emergence of multiple beneficial mutations have long attracted interest, but opportunities to observe these dynamics empirically are rare—exceedingly so in wild populations. This limitation does not exist for the Hawaiian field cricket populations we studied. Under extreme selection against song, two different adaptive song-loss phenotypes, Fw and Cw, have repeatedly spread in the same populations. We find these phenotypes have nonoverlapping genetic architectures and are frequently coexpressed. While it is plausible that these mutations will fix in future generations, our findings indicate their mutual co-occurrence has substantially slowed this adaptive proliferation and impeded the loss of singing males, despite extreme negative selection on male song.

Adaptive mutations are expected to be very rare (48). This does not appear to have been the case among typically small and fragmented Hawaiian populations of *T. oceanicus* evolving under fatal parasitism by *O. ochracea*, across which four different and apparently novel song loss ‘morphs’ have emerged within the last 20 years (17, 21, 22). On top of these divergent wing morphs, superficially similar flatwing phenotypes have apparently arisen independently on at least three occasions (13, 18). The reason for this exceptional proliferation of adaptive forms is clear. The introduction of *O. ochracea* to Hawaiian populations of *T. oceanicus* radically changed the fitness landscape, from one in which male singing ability was strongly favored by benefits in attracting female mates, to one in which any mutation that corrupts a male’s ability to sing offers a selective advantage. In addition, acoustic signaling is likely a very large mutational target due to the many behavioral, physiological, and morphological loss-of-function mutations that might disrupt it. While this scenario of similar adaptive mutations competing within a population might be presumed to be rare, we point to the widely observed potential for similarly adaptive phenotypes to emerge and spread across populations and species [i.e., parallel, convergent, or repeated evolution (49)], at least some of the time through different genetic mutations (11). Thus, the opportunity for alternative adaptive mutations to be introduced to the same populations through gene flow or de novo mutation might be underappreciated. Repeated evolution of similar adaptations is more likely under strong selection, which emphasizes the potential importance of selective interference under such conditions.

The maintenance of nonadaptive variation underlying singing ability in male crickets is of particular evolutionary importance because silent male *T. oceanicus* benefit from the retention of singing males, which they rely upon to adopt satellite mating tactics (17). Cricket song thus plays an important role in the social environment, and also affects traits such as adult reproductive investment (50), neural gene expression (44), and locomotive activity (51). Should selection against song decrease in severity in populations in which Cw and Fw co-occurrence has impeded the loss of singing-capable WtNw male phenotypes, the latter would be expected to spread through the population. Males would thus regain the ability to attract female mates, without requiring the secondary evolution of song or other signaling modalities (52, 53). Additionally, Fw and Cw phenotypes are associated, though not causally, with very large linked regions that appear to be chromosomal inversions. These linked regions are polymorphic in all three populations, and it is plausible that their statistical cosegregation with competing Cw and Fw mutations has contributed to the persistence of these substantial sources of genetic variation.

Standing genetic variation is of central importance to the ability of wild populations to adapt under extreme selection, particularly when selection is strong and adaptation must occur quickly (54). However, selective sweeps erode genetic variation, potentially reducing a population’s potential to adapt to future selection. In wild populations of *T. oceanicus*, we find phenotypic and genetic variation in the form of multiple, contrasting wing morphologies is maintained despite extreme selection. This maintenance of variation in the face of strong selection is due, at least in part, to nonadditive fitness benefits of interacting adaptations impeding their spread. Our findings demonstrate that the interaction between selection and genetic variation can be unpredictable, and highlight distinctive evolutionary dynamics when similar adaptations co-occur.

## Materials and Methods

Further details of methods are provided in supporting information.

### Heritability Crosses.

We performed half-sibling crosses between 37 males and 111 females, for each of the four possible combinations of parental wing phenotype (Cw/Wt) × parental sex (M/F). Each male was sequentially paired with three females, for five days each, to produce up to three half-sib F_1_ families. Offspring from each cross were reared to adulthood and phenotyped. Results were analyzed using generalized linear mixed models in R v4.0.2 (55).

### Genetic Mapping.

We performed restriction site-associated DNA sequencing to obtain sequences of single-end 100 bp reads distributed across the genome for 380 individuals (376 F_2_ offspring plus F_0_ and F_1_ parents). Library preparation and sequencing on the Illumina HiSeq 2000 platform to produce single-end 100 bp reads was performed by Floragenex (Oregon).

### Whole Genome Sequencing Data.

We extracted and sequenced DNA from 90 males from three populations: Oahu.CC, Kauai.CG, and Hawaii.UH. (30 per population) on an Illumina NovaSeq platform using S4 chemistry, generating 2 × 150 bp reads. Library preparation, sequencing, and trimming were performed by the Centre for Genomic Research at the University of Liverpool. Association tests were performed using linear models, with significance tested via likelihood ratio tests (56).

### RNA Sampling.

We sampled RNA from final instar forewing buds from a polymorphic full-sib family. Two wingbuds from different crickets were pooled per RNA sample. Sixteen libraries were prepared and sequenced on an Illumina NovaSeq platform using S1 chemistry to produce 2 × 150 bp reads by the Centre for Genomic Research, University of Liverpool.

### Mate preference trials.

Virgin males and females were isolated from the Oahu.CC stock population at the final instar. Individuals were phenotyped and used in mate preference trials at 5 to 10 d postadult eclosion. Trials were conducted within a 210 × 230 mm arena together under red light. Trial data were analyzed using GLMs with binomial error distribution.

### Life history assays.

We recorded survival, structural size (pronotum length), and wet mass for 91 males and 48 females from a mixed stock population. Analysis of female mass across adulthood was performed using a linear mixed model using *lme4* (57). Male mass did not vary significantly with age, so was analyzed at 14-d adulthood. Survival was analyzed using Cox proportional hazards regression in the R package survival (58).

## Supplementary Material

Appendix 01 (PDF)

## Data Availability

Previously unpublished sequencing whole genome, RNA- and RAD-sequencing data are available in the NCBI SRA under BioProject PRJNA1019311 (59). Other data supporting this publication are available at https://doi.org/10.13016/ehst-57yz (60). Scripts used for processing and analyzing data are available at https://github.com/jackgrayner/competing_adaptations (61).
