# Supplementary material for: Competing adaptations maintain nonadaptive variation in a wild cricket population
Source: Proc Natl Acad Sci U S A. 2024 Aug 1;121(32):e2317879121. doi: 10.1073/pnas.2317879121 (PMC11317585; doi:10.1073/pnas.2317879121)
Supplement: Supplementary file 1 — Appendix 01 (PDF) [file pnas.2317879121.sapp.pdf]

Supporting Information for

Competing adaptations maintain non-adaptive variation in a wild cricket population

Jack G. Rayner, Franca Eichenberger, Jessica V. A. Bainbridge, Shangzhe Zhang, Xiao Zhang, Leeban H. Yusuf, Susan Balenger, Oscar E. Gaggiotti, & Nathan W. Bailey

Corresponding authors: Jack G. Rayner, Nathan W. Bailey

Email: jackgrayner@gmail.com, nwb3@st-andrews.ac.uk

This PDF file includes:

Supporting text

Figures S1 to S19

Tables S1 to S6

SI References

SUPPORTING METHODS

Cricket rearing and husbandry

Unless otherwise indicated, analyses relate to samples derived, or collected, from a wild population at a Community Center in Manoa, Oahu (Lat.: 21.316219, Long.: -157.809922, Fig. 1B). This is the population in which we first observed the Cw phenotype in 2017. Since it was first observed, Cw has been stably present at approximately 50% frequency in the Community Center population (personal observations, JGR & NWB). We also include whole genome sequencing samples from two more populations in which Cw and/or Fw phenotypes also co-occur: the Kilauea Common Ground on the island of Kauai (Lat.: 22.197576, Long.: -159.417606), the University of Hawaii Hilo campus on the island of Hawaii (Lat.: 19.703186, Long.: -155.080237).

Laboratory experiments and trials were performed using a stock population, or inbred populations produced from the stock population, derived from eggs of wild caught *T. oceanicus* females captured in 2017 from a location in Manoa, Oahu, HI. Eggs from wild-caught females were transported back to the University of St Andrews, UK, and were reared as a mixed stock population across multiple 20L boxes at 25c, on a 12:12 photoreversed light:dark cycle. Crickets were fed Purina Rabbit Chow (Burgess Excel) and provided with cotton pads moistened with distilled water (both as a water source, and for oviposition), as well as cardboard shelter.

Heritability crosses

We performed half-sibling crosses between 37 males and 111 females, for each of the four possible combinations of parental wing phenotype (Cw/Wt) × parental sex (M/F). Each male was sequentially paired with three females, for five days each, to produce up to three half-sib F_1 families. In each of these combinations a mix of normal-wing and flatwing male phenotypes were included, with female genotype at the *Flatwing* locus unknown. Offspring number was recorded 30 days after hatching for each pairing that produced offspring, and where necessary numbers were then reduced by culling to avoid overcrowding (<~150 juvenile crickets per 20L box). The curliness of each parental male and female's wings was scored according to a qualitative scale (0-4 per wing; Fig. S2; Table S1).

Offspring from each of the successful crosses were reared to adulthood and the curliness of each of their wings was similarly scored on discrete (Cw vs. Wt) and qualitative scales. We aimed to record the phenotype of >=15 of each sex from each full-sib F_1 family. From one of these F_1 families, five male/female pairings with contrasting curliness scores (one Wt, one highly Cw) were removed and paired to produce inbred F_2 families, one of which was used for association mapping, and another which was used for RNA-seq analysis.

Results of crosses were analysed using generalised linear mixed models with a response variable of the proportion of Cw offspring, separated by offspring sex. We first ran a linear mixed model including only random intercepts for maternal ID nested within paternal ID, i.e. (1| father/mother), to assess heritability. Subsequent models also included fixed effects of rearing density in the first 30 days of development, offspring sex, and an interaction between paternal and maternal Cw phenotypes to test for parent-of-origin effects. We also ran the same models using a predictor of mean curliness score per family, rather than the proportion of Cw offspring. Models were fit using the R package *lme4*, tested using type II or III Wald's Chi-square tests (depending on whether an interaction was included) implemented in the R package *car*, and R-squared values for fixed and random effects were estimated using the R package *MuMIn* (1).

Extraction of nucleic acid samples

Purified DNA and RNA samples were extracted as previously described in Zhang et al. (2021). Briefly, a CTAB/chloroform-based extraction protocol was used to extract DNA from neural or leg tissues. RNA samples were extracted using a Trizol/chloroform-based protocol, with subsequent washes performed using filter columns following the ThermoFisher Purelink protocol.

Genetic mapping

We performed restriction site associated DNA sequencing (RAD-seq) to obtain sequences of single-end 100bp reads distributed across the genome, using the SbfI restriction enzyme. A total of 380 individuals were sequenced: two F_0 full-sib parents, two F_1 full-sib offspring, and 376 inbred F_2 offspring (197 Wt, 178 Cw). We sequenced the 376 samples to ~30x, and as part of a pilot study also sequenced a further plate at higher depth (~100x), which included the F0 and F1 samples. Library preparation and sequencing on the Illumina HiSeq 2000 platform to produce single-end 100bp reads was performed by Floragenex (Oregon, USA).

Stacks (v 2.6.0) was used to demultiplex libraries, and BWA-mem (v 0.7.17) (2) to align the sequences to the *T. oceanicus* reference genome (Zhang et al. in review) with default parameters. Variant discovery was performed using *gstacks* (3), with a minimum mapping quality score of >=20. The resulting catalog was filtered to retain only SNPs with a minor allele frequency > 0.1 and genotyped in > three-quarters of samples in both phenotypes, then converted to vcf format. Genotypes with quality scores less than 20 were filtered using vcftools (v 0.1.16) (4). We rescored X-linked heterozygote calls for each of the male samples as homozygote for the dominant allele if a binomial test revealed significant ($P<0.05$) differences in allelic depth, and removed heterozygote calls without significant allelic imbalance. We removed genotype calls at loci with sequencing depth < one-third or > three-times the respective sample's mean sequencing depth, atop a fixed minimum sequencing depth of 10x. Sequencing depth filters were halved for X-linked loci in male samples. Because the presence of PCR duplicates in single-end RAD-seq data is expected to exaggerate heterozygosity, all heterozygotes with strong skew in allelic depth (binomial test P-value < 0.01) were rescored as homozygotes. Association tests between SNPs and Cw presence/absence were performed using Fisher's exact tests in PLINK (v 1.9) (5), discarding any loci missing in >= 30% of samples, and samples lacking genotypes at >= 60% of loci. P-values were adjusted for multiple testing using Bonferroni correction. A small number (N = 3) of highly significantly Cw-associated SNPs located on Chr4 were removed from the analysis, because visualisation of linkage patterns indicated they showed much stronger linkage with the 0:80 MB region of Chr2 than nearby regions of Chr4, indicating these SNPs were in fact located on Chr2.

RNA sampling

We sampled RNA from developing forewing wing buds from a polymorphic F_2 full-sib family. This F_2 family resulted from a different F_1 cross than that used for the generation of RAD-seq data, though it was derived from the same F_1 full-sib family. We sampled wing buds at the final instar, immediately prior to adult eclosion, two days after the penultimate moult. We sampled at this stage because curly-wing morphology is not yet externally visible, whereas it is immediately visible following the final adult moult. Thus, at the time of sampling we were unaware of each male's adult phenotype, so we sampled only one developing (dorsal-right) wing bud and recorded the phenotype of the other wing after adult eclosion.

We retained only unambiguous Cw and Wt phenotypes. Two wing buds from different crickets were pooled per library. For Cw individuals, we retained only males assigned curliness scores of 2 or 3 for their left wing. For each pooled Cw sample, one male had a curliness score of 2 and the other a curliness score of 3. Flatwing was polymorphic in the inbred F_2 family used for sampling wings, so we included both male wing vein phenotypes to produce 4 mRNA libraries for each combination of Wt/Cw x Nw/Fw. RNA sampling was performed under CO_2 anaesthesia to minimise effects of surgical wing bud removal on welfare and gene expression, and developing wing tissue was removed using dissection scissors. Wing tissues were submerged in RNAlater and stored at -20° C.

RNA-seq processing and differential expression analysis

mRNA libraries were prepared using PolyA selection and sequenced on an Illumina NovaSeq S1 to produce paired 150bp reads. Reads were provided after trimming adapter and low quality sequences by the Centre for Genomic Research, University of Liverpool. Quality trimming was performed using Sickle v1.200 (https://github.com/najoshi/sickle/releases/tag/v1.2) with a minimum window quality score of 20, removing reads shorter than 15bp. Filtered reads were aligned to the genome using HISAT2 (6), then individual transcriptomes assembled and merged using Stringtie (7).

Transcript abundances were quantified at the ‘gene’ level. We filtered any genes not expressed at greater than one count-per-million in at least four samples.

Differential expression analysis was performed in DEseq2 (8), with models tested using likelihood ratio tests and an FDR-adjusted P threshold of 0.05 for a gene to be considered significantly differentially expressed. Visualisation of gene expression patterns revealed that two samples, one CwFw and one CwNw male, showed gene expression patterns dissimilar with the other samples, however removal of these samples did not produce results with different interpretation, and there was a strong overlap (100% of genes DE between Cw/Wt phenotype in the reduced dataset were also DE in the complete dataset), so we opted to retain the full dataset for our analysis. Overrepresentation of GO terms was tested using *Drosophila melanogaster* blastx hits for DE genes against all genes present in the filtered transcriptome (e-value < 1e-6) in PANTHER (9).

Whole genome sequencing data

To further investigate candidate Cw-associated regions from the RAD-seq data, we used whole genome sequencing data at ca. 20X depth obtained from leg tissue samples of wild-caught individuals by Zhang et al. (2021). We aligned reads to the genome using bwa-mem2 with default parameters, then removed secondary alignments and PCR duplicates as in Zhang et al. (2021). We used bcftools (10) to call variants on Chr2, which were filtered with vcftools to remove genotypes with sequencing depth < 10 or > 120, mapping quality < 20, genotype quality < 20, and minor allele frequency < 0.05. We also called variants on the X chromosome as above, but specifying samples were haploid, and with a lower maximum sequencing depth of 70. We used PLINK (v 1.9) to perform principal component analysis after merging the vcf for these samples with the vcf for the RAD-seq samples. To quantify linkage, we first thinned the filtered vcf using vcftools to retain 1% of variants (--thin 0.01). The resulting data were converted to .PED format using PLINK and used as input for linkage disequilibrium analysis in Haploview v4.2 (11).

We also collected whole genome sequencing data from hindleg samples collected in 2021/2022 from the same population as above (Oahu.CC), as well as two other populations (Kauai.CG and Hawaii.UH) in which we observe Cw phenotypes. For these samples, we had recorded whether each individual visibly expressed the curly-wing phenotype. We extracted genomic DNA from and sequenced 90 males (30 from each population) on an Illumina NovaSeq S4, generating 2x150 bp reads at ca. 15X average depth. Library preparation, sequencing, and trimming of adapter and low quality sequences was performed by the Centre for Genomic Research at the University of Liverpool. Quality trimming was performed using Sickle v1.200 (https://github.com/najoshi/sickle/releases/tag/v1.2) with a minimum window quality score of 20, removing reads shorter than 15 bp. We aligned reads to the *T. oceanicus* genome V2 with bwa-mem2 and default parameters, and marked PCR duplicates using PicardTools (Broad Institute, 2019). We identified and called variants on Chr2 for each population using bcftools, filtered with vcftools to remove genotypes with sequencing depth < 5 or > 100, mapping quality < 20, genotype quality < 20, and minor allele frequency < 0.1. Linkage was calculated in Haploview after thinning the VCF to retain 0.1% of SNPs. VCFs for each of the populations were merged using bcftools, and converted to BED format using Plink. PCA was performed with PLINK v1.9, excluding SNPs with minor allele frequency < 0.15 and which were not genotyped in >= 80% of samples. Association tests were performed using linear models, with significance tested via likelihood ratio tests, in GEMMA (12). These included a covariate of population ID, and excluded positions genotyped in less 90% of samples, and we filtered SNPs with minor allele frequency < 0.15.

Variant calling, linkage, and association tests between Nw and Fw phenotypes were performed as above, except restricting variant calling to the X chromosome, specifying that all samples were haploid (as males only carry a single X), and filtering for a maximum sequencing depth of 70. For the Oahu.CC population, we used bcftools merge to combine data collected in the current study (N=30 samples) with that previously collected by Zhang et al. (2021) in 2017, for a total sample of 50. We performed association tests separately for each population, as previous research suggests the genetic basis of Fw differs between populations (13, 14). We filtered SNPs with an allele frequency < 0.2.

Mate preference trials

Males and females (83 of each sex) were isolated from the Oahu.CC stock population at the final instar prior to adult eclosion to ensure virginity, and separately reared in 113 ml deli pots. Cw and Fw phenotypes are both present and polymorphic in this population. Individuals were phenotyped at adulthood, and used in mate preference trials at 5-10 days post-adult eclosion, when both sexes are reproductively mature. To conduct trials, a male and female were chosen at random and placed in empty upturned 113ml deli pots within a 210 × 230 mm arena together under red light, given three minutes to acclimate, then the deli pots were removed and trials were run for 10 minutes after one of the two began to move (unless they did not move within 2 minutes, in which case the trial was not included in analysis). Trials were filmed on a Nikon D3300 digital camera. We recorded whether the male produced (or attempted to produce) courtship song, and whether the female mounted the male within the 10 minute trial.

Trial data were analysed using GLMs with binomial error distribution in R v4.0.2 (R Core Team 2020): female mount (Y or N) ~ male court (Y or N) × male morph (WtNw, Fw [CwFw & WtFw pooled due to small sample size], or CwNw). Binomial GLMs were checked for overdispersion and tested using type III Wald's Chi-squared tests. Although we originally used each male and female in multiple trials (up to four times), having prevented them from spermatophore transfer during trials to ensure virginity, attempts to include male and female ID in the model as random effect terms using *lme4* (16) produced convergence errors, so we used only the first trial for each male and female to avoid pseudoreplication and confounding effects of prior experience in our analyses.

Life history assays

To test for correlated fitness effects of Cw in non-wing tissues, we recorded survival as well as structural size (pronotum length to nearest .1 mm) and wet mass (to the nearest .01 mg) at adulthood for 91 males and 48 females from a mixed stock population. All individuals were separated upon reaching their final instar and reared individually without access to the opposite sex (i.e., virgins) in 113 ml deli pots with food and water available *ad libitum*, as well as cardboard shelter. Mass and survival status were recorded every 7 days up to 91 days post-adulthood in females and 84 days post-adulthood in males. While mass can vary with age, pronotum length is fixed at adult eclosion. Therefore, we measured pronotum length 3 times and used the mean average to account for measurement error. All crickets were virgin. We used pronotum length and mass measures to calculate scaled mass index (SMI) separately for each sex, a measure used to estimate body condition (17). Survival was recorded up to a maximum of 91 days in females, and 84 days in males (due to differences in eclosion time).

We used mass measurements to examine adult size and scaled mass index to examine body condition; the latter incorporating information about both mass and structural body size (pronotum length) (17). Analysis of female mass across adult lifespan was performed using a linear mixed model using *lme4* in R, including linear and quadratic terms for adult age in days, Wt/Cw phenotype, and interactions. For males, there was no evidence of a relationship between age and mass, so we analysed mass at 14-days post-eclosion, an age at which males are reproductively active but do not usually show signs of senescence. We used a linear model with predictor terms of wing shape (Wt/Cw) and wing venation (Nw/Fw), and removed the interaction between the two because it did not approach statistical significance. Note that Fw/Nw could not be phenotyped in females as Fw expression is male-limited. For models with and without interactions we used Type II and III Wald's Chi square tests, respectively, using the *car* package in R.

Lifetime survival probability was analysed using Cox proportional hazards regression in the R package *survival* (18). We included predictors of wing shape and SMI for females, and wing shape, wing venation and SMI for males. Interactions that did not approach significance were removed from the model.

Simulations

Simulations were run using SLiM v4.0.1 (19). Adaptive mutations, one autosomal and one X-linked, were introduced to the maternally inherited genome of 5 random males in a population of 500 diploid individuals with a 50:50 sex ratio. Simulations were run for 500 generations, with 10,000 runs per scenario. Fitness benefits of the two mutations were male-limited, being neutral in females, and varied across scenarios. Whenever a single mutation was completely expressed (i.e., homozygous, hemizygous, or heterozygous for a mutation with a dominance coefficient of 1), males had relative fitness 1.3 (i.e., a +0.3 fitness benefit). When both mutations were completely expressed by the same individual, the individual had a fitness benefit of 1.6 in the *additive* scenario (where individuals expressing both mutations had greater fitness than individuals expressing either one), 1.3 in the *non-additive* scenario (where individuals expressing both mutations had no advantage over individuals expressing either one), and 1.2 in the *negative* scenario (where individuals expressing both mutations were disadvantaged relative to individuals expressing either one).

If the dominance coefficient (h) for the autosomal mutation was < 1, and the autosomal mutation was heterozygous, the fitness benefit was modified accordingly. Thus, males who were heterozygous for the autosomal mutation had a fitness of ($1 + 0.3h$). Moreover, in the *additive* scenario, males heterozygous for the autosomal mutation had fitness ($1.3 + 0.3h$) if they also carried the X-linked mutation (which was always expressed and conferred a fitness benefit of + 0.3). In the *non-additive* scenario, males who were heterozygous for the autosomal mutation had a fitness 1.3 if they also carried the X-linked mutation – as the X-linked mutation was always expressed, and the mutations did not have additive effects on fitness. In the negative scenario, males who were heterozygous for the autosomal mutation had a fitness of ($1.3 - 0.1h$) if they also carried the X-linked mutation, with 0.1 the fitness cost of completely expressing both mutations relative to expressing either one.

Supporting figures

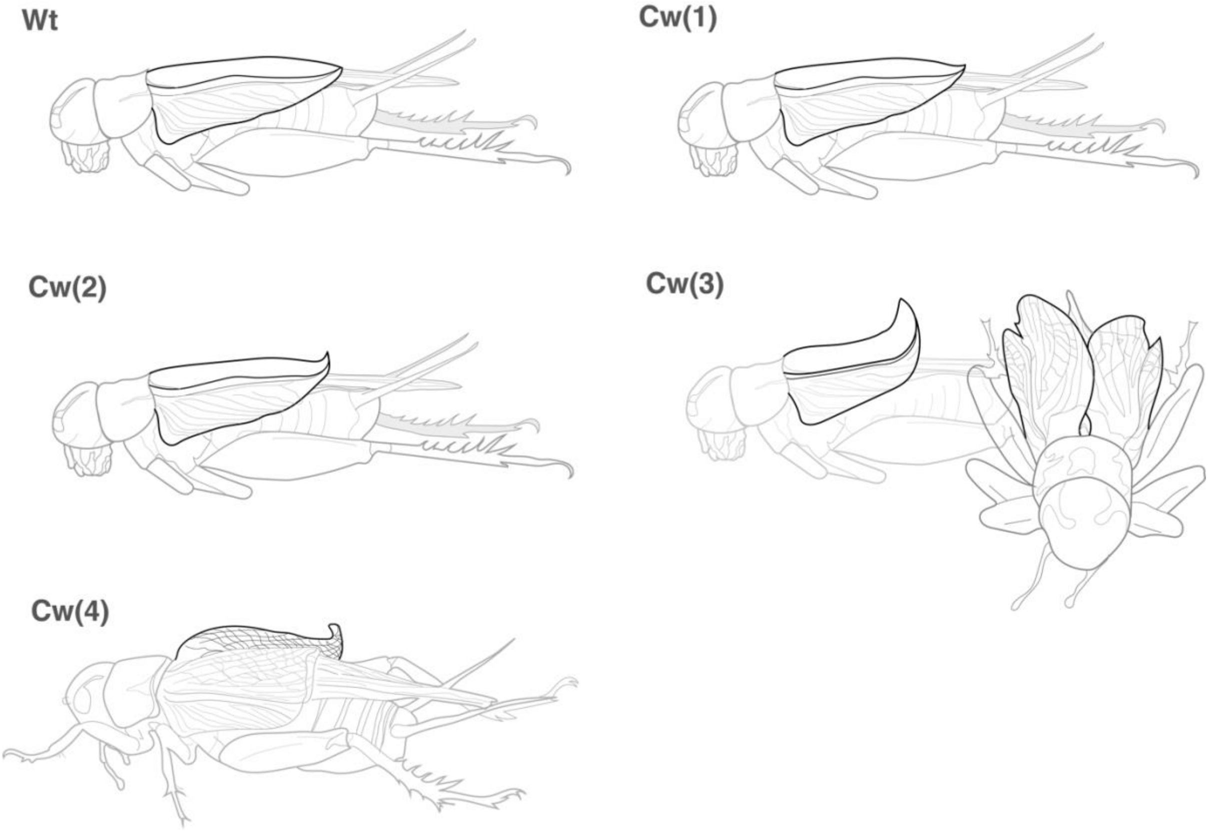

Figure S1. Illustrations of Wt and Cw morphology, with examples of wings with curliness scores of 1 (slight) to 4 (extreme).

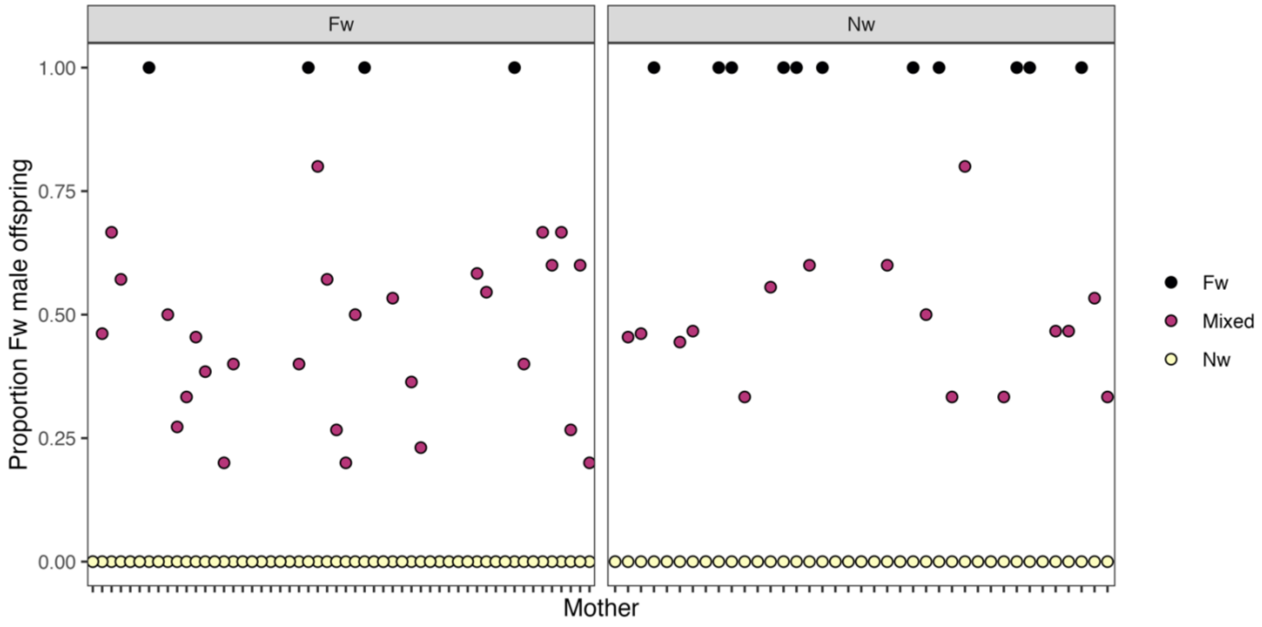

Figure S2. Proportions of Fw male offspring across families. Fw tended to be present at 0%, 100% or ca. 50% frequencies, consistent with its known single-locus, X-linked mode of inheritance. Panels show results of crosses involving Fw and Nw fathers, showing no greater tendency for Fw fathers to produce Fw offspring: as expected in the case of X-linkage, in which males do not transmit a copy of the X-chromosome to male offspring.

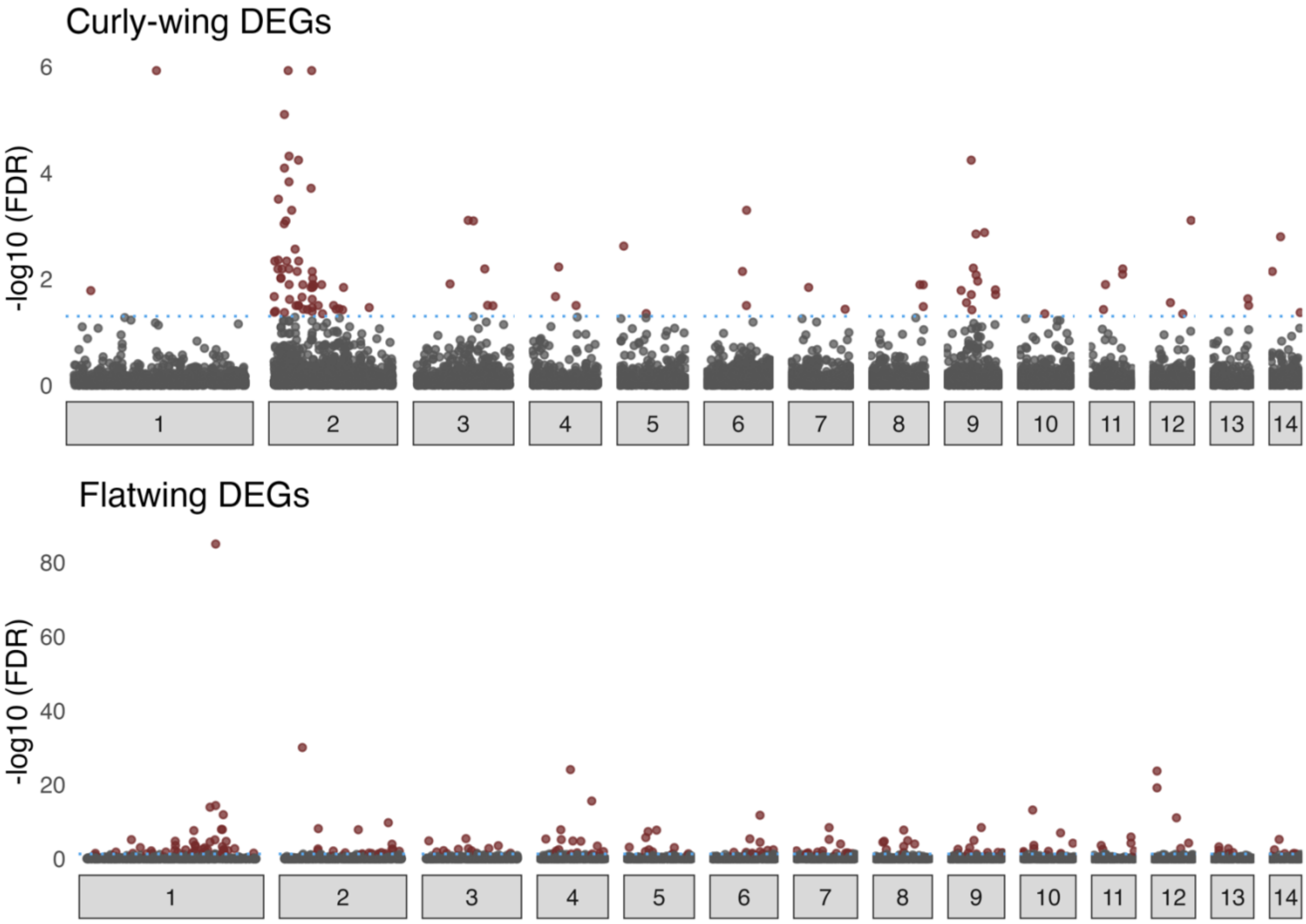

Figure S3. Locations and relative significance of genes DE between Wt and Cw phenotypes, for which the causative region is on chromosome 2, and Nw and Fw phenotypes, for which the causative region is located on the X chromosome. Note the order of magnitude difference in Y-axis scales between B and C. Points are coloured by statistical significance (FDR<0.05) also indicated by the dotted horizontal line.

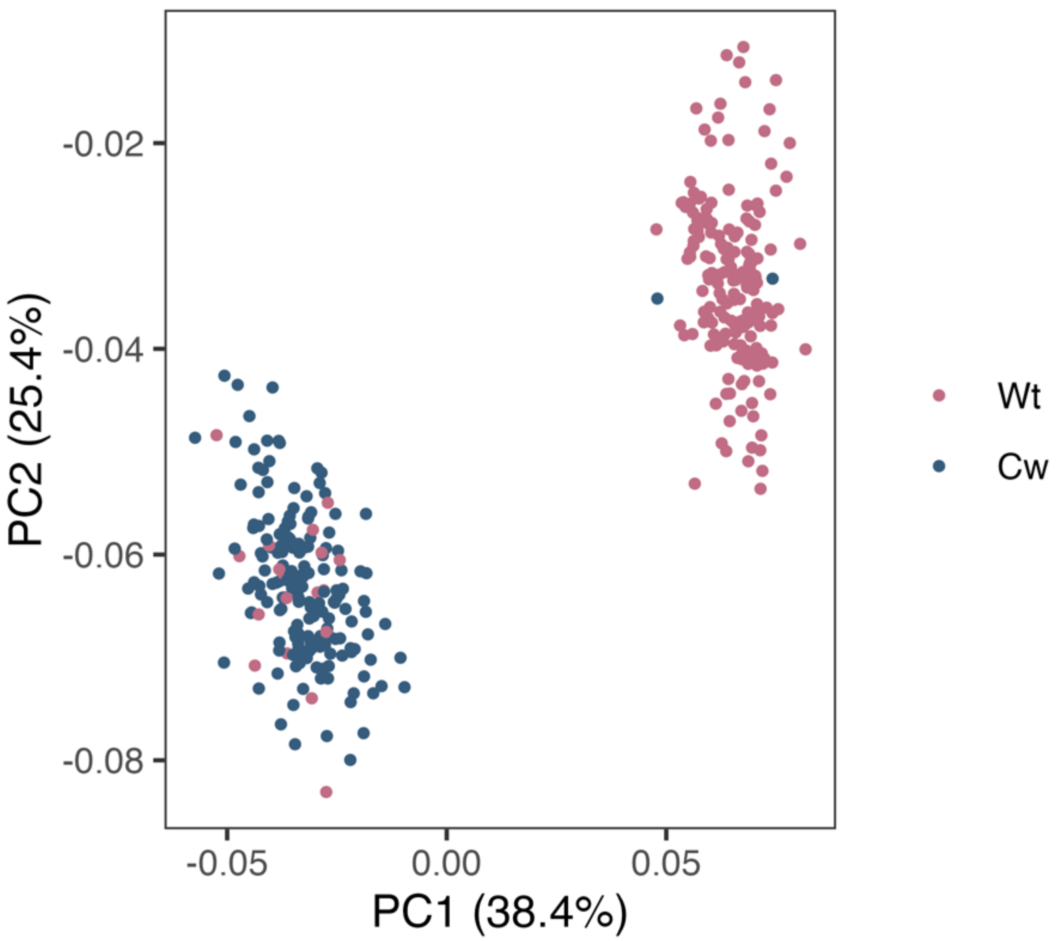

Figure S4. PCA generated using RAD-seq data from the Cw mapping family, including SNPs from the region of 0 to 80 MB on chromosome 2. We observe two discrete clusters of samples, rather than the three expected in the case of an inversion (cf. Fig. S5 and Fig 3A). This suggests that the Cw parent in our F1 cross was heterozygous for the Cw-associated inversion, resulting in offspring that were either homozgyous for the non-inverted copy, or heterozygous.

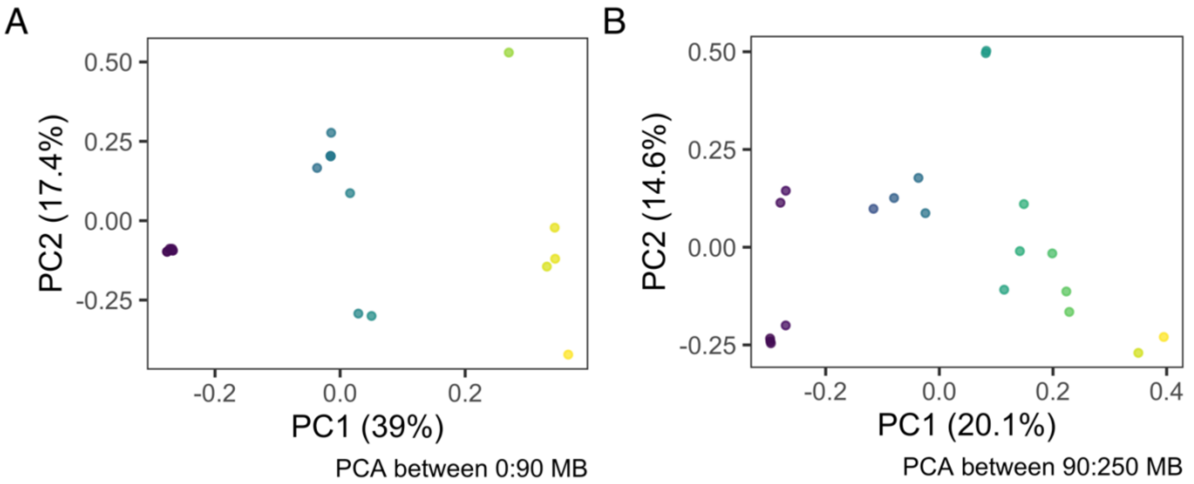

Figure S5. PCA of variants on Chr2 using data from Zhang et al. (2021) (13)**.** Plots are split into A) the region surrounding the inversion (0:90 Mb), and, B), the rest of Chr2. Points in each plot are coloured based on values on PC1, revealing discrete grouping of haplotypes in the region of the inversion but not along the remainder of the chromosome.

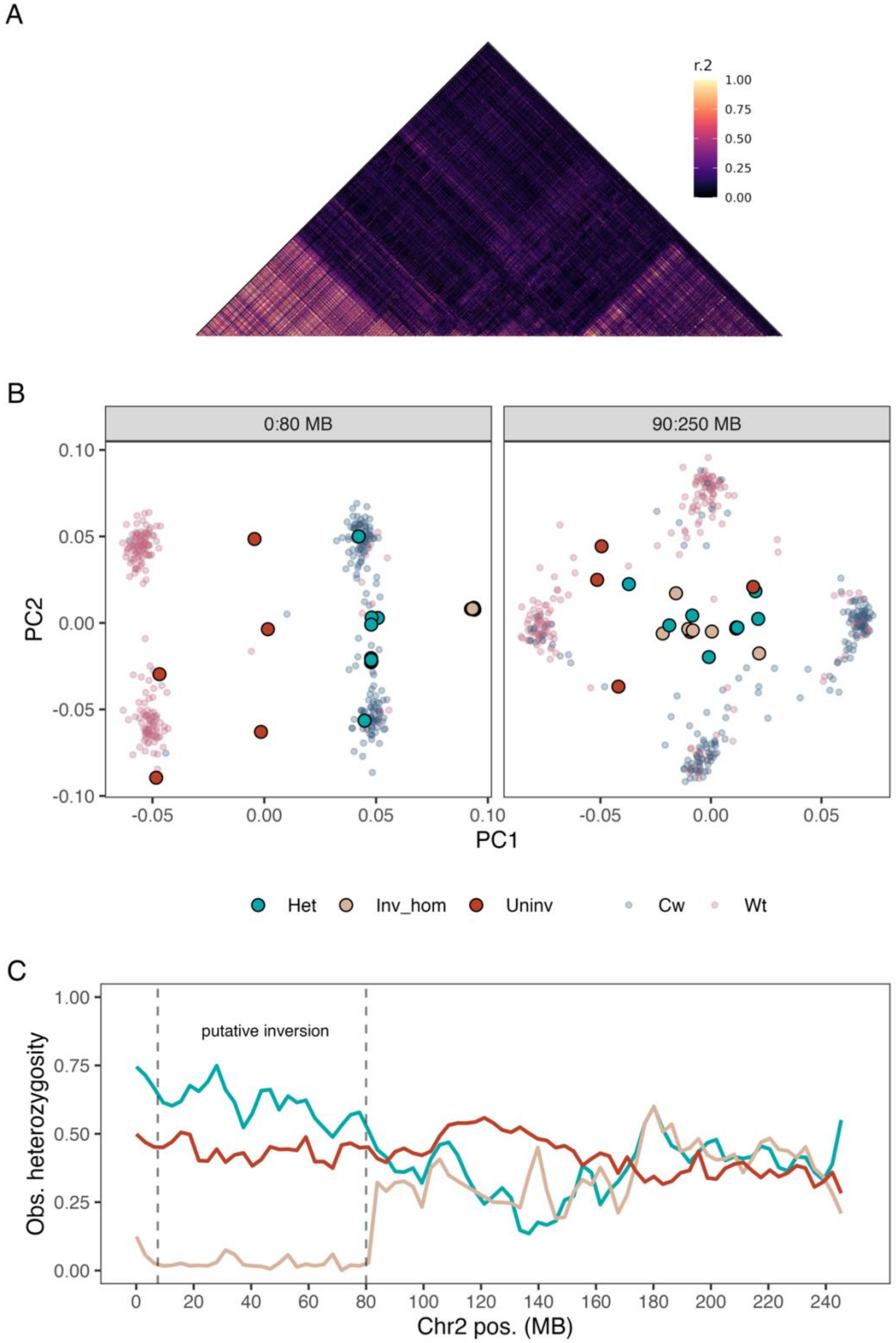

Figure S6. Evidence of a large inversion in the region of Chr2 associated with curly-wing. (A) Strong linkage between SNPs in the region of the candidate inversion on Chr2. **(B)** PCA incorporating RAD and WGS data for variants present in both filtered datasets on Chr2, including SNPS within the Cw-associated region identified by the RAD-seq analysis (left), and across the remainder of the chromosome (right). Small circles show RAD-seq samples coloured by phenotype, whereas larger filled points show WGS samples, filled by putative genotype with respect to the candidate inversion.

Note that wild samples in the putative non-inverted cluster show much greater variability on PC1, with respect to wild samples carrying the inversion, as expected following the reduction in genetic diversity associated with a chromosomal inversion. **(C)** Heterozygosity on Chr2 between samples from the three putative genotypes, with the candidate inversion highlighted with vertical dashed lines bracketing the first 80 Mb. Solid lines show LOESS trended means.

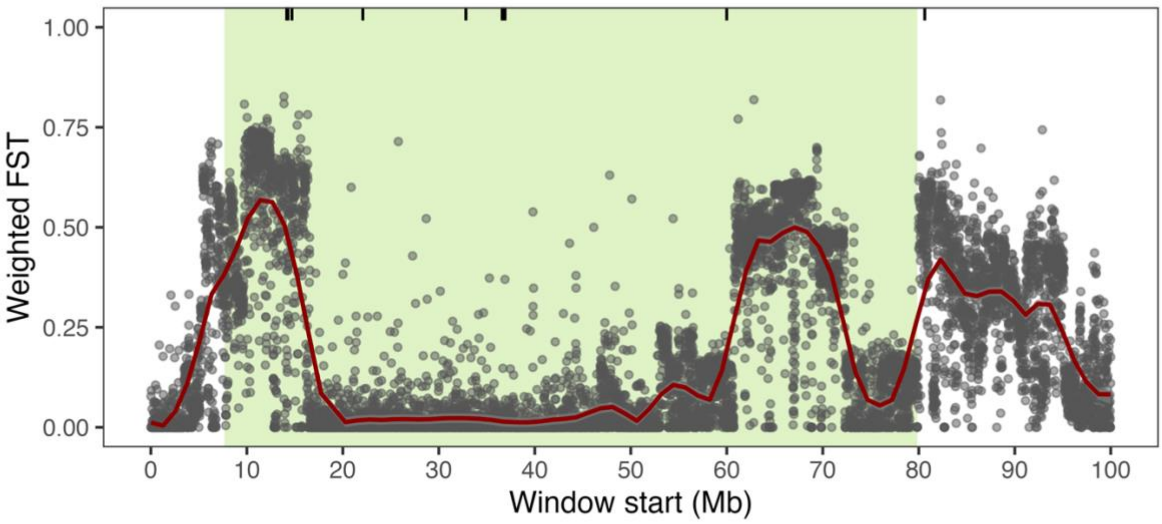

Figure S7. F_{ST} values in 10kb windows with 10kb step size, between samples from Hawaii.UH and Kauai.CG that clustered together on PC1 but had opposite phenotypes (see Fig. 3A). The red line illustrates LOESS regression. The three regions showing elevated F_{ST} thus present candidate regions for variants underlying the Cw phenotype. The green area shows the inferred region of the putative inversion, and tick marks indicate the locations of genes annotated for involvement in serine-type endopeptidase inhibitor activity.

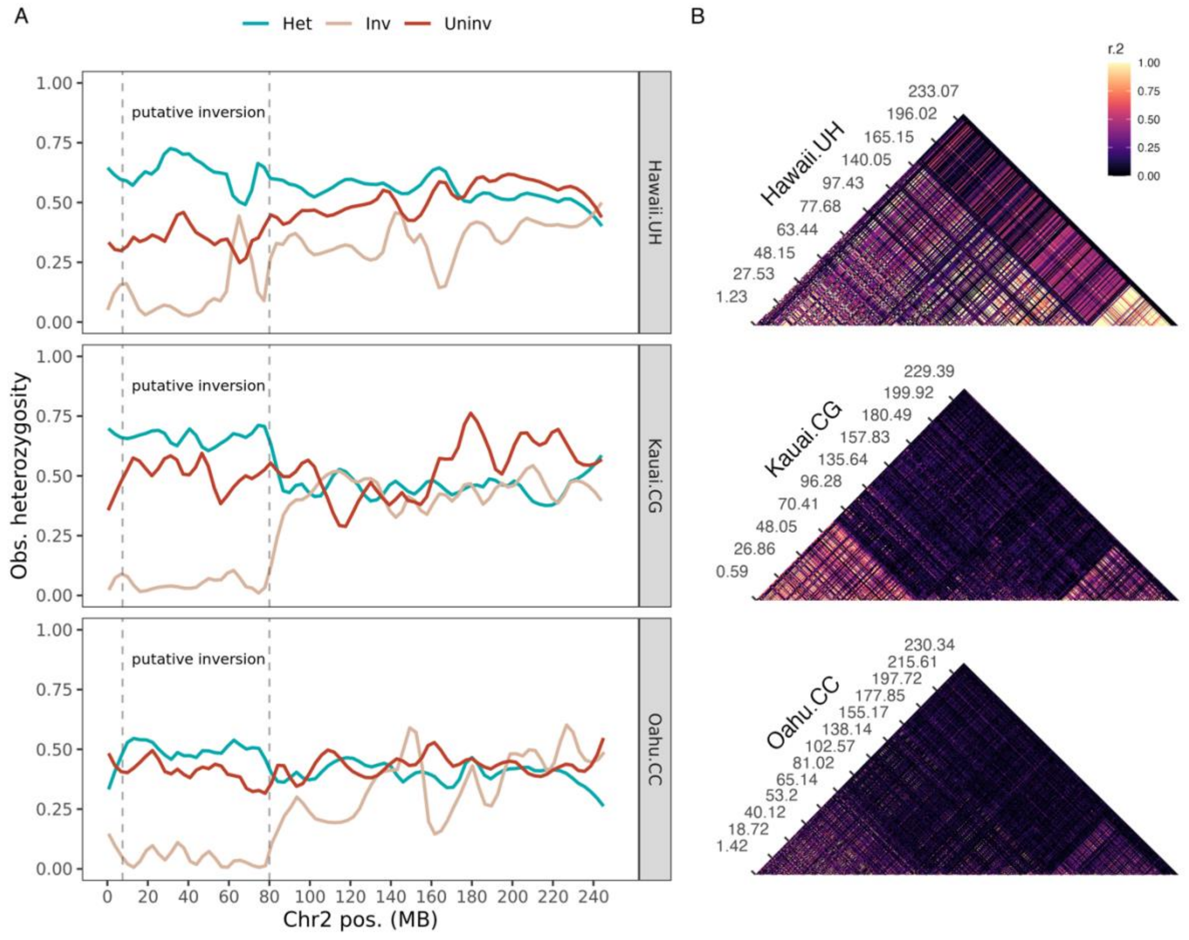

Figure S8. A) Heterozygosity between inferred genotypes (based on PC1 values from Fig 4A) across the putative inverted region of 7.5:80 Mb on Chr2, in three populations. **B**) Linkage between thinned (0.0005) SNPs genotyped in >50% of samples across all three populations.

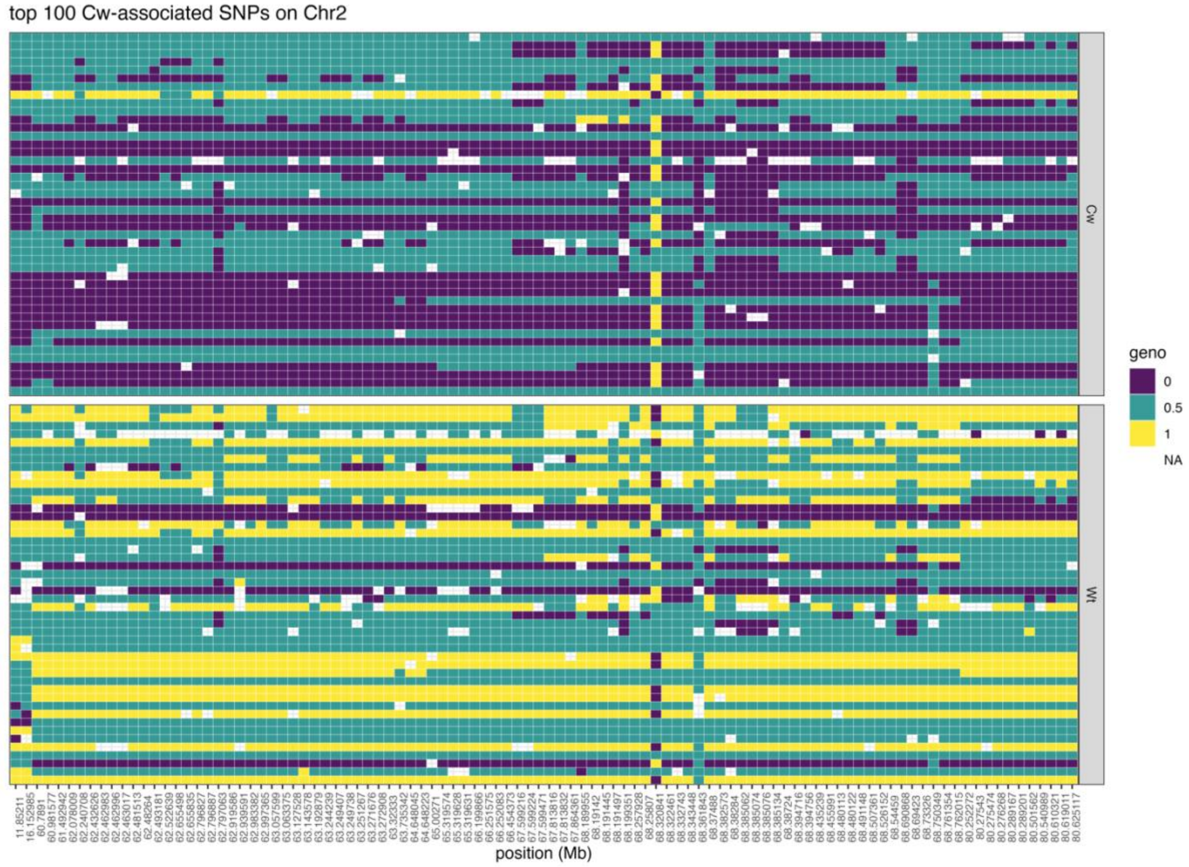

Figure S9. Genotypes (0=homozygous major allele; 0.5=heterozygote; 1=homozygous minor allele) across samples for each of the top 100 Cw-associated SNPs.

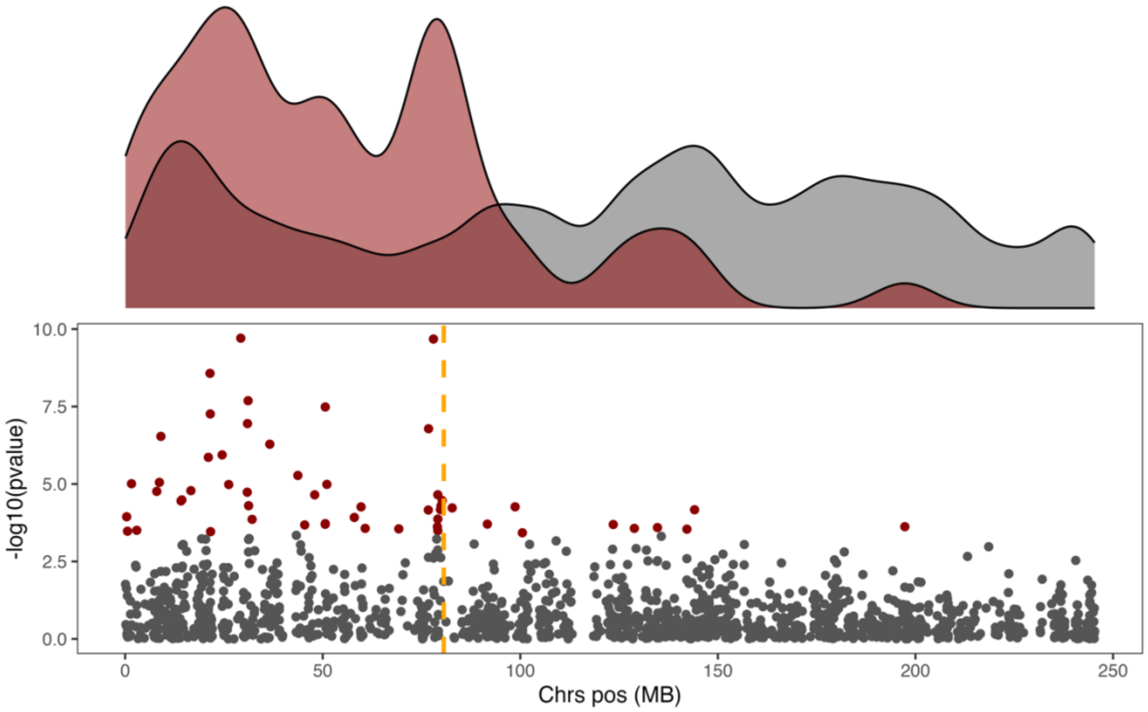

Figure S10. Density and significance of expressed genes along Chr2, with DE_{Cw} genes shown in dark red. The vertical orange dashed line shows the position of *ITIH4.*

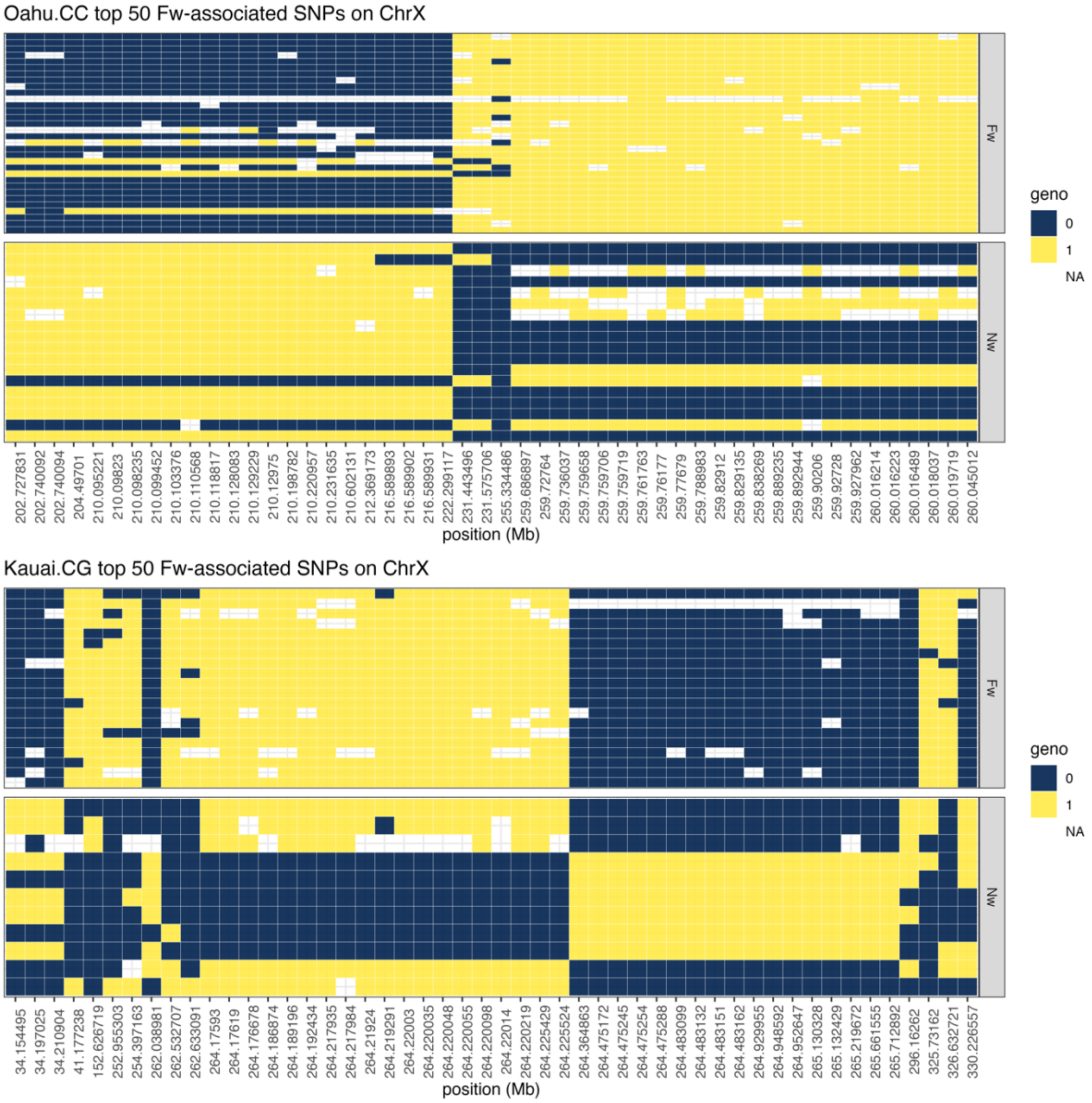

Figure S11. Genotypes across samples for each of the top 50 Fw-associated SNPS for Oahu.CC and Kauai.CG populations.

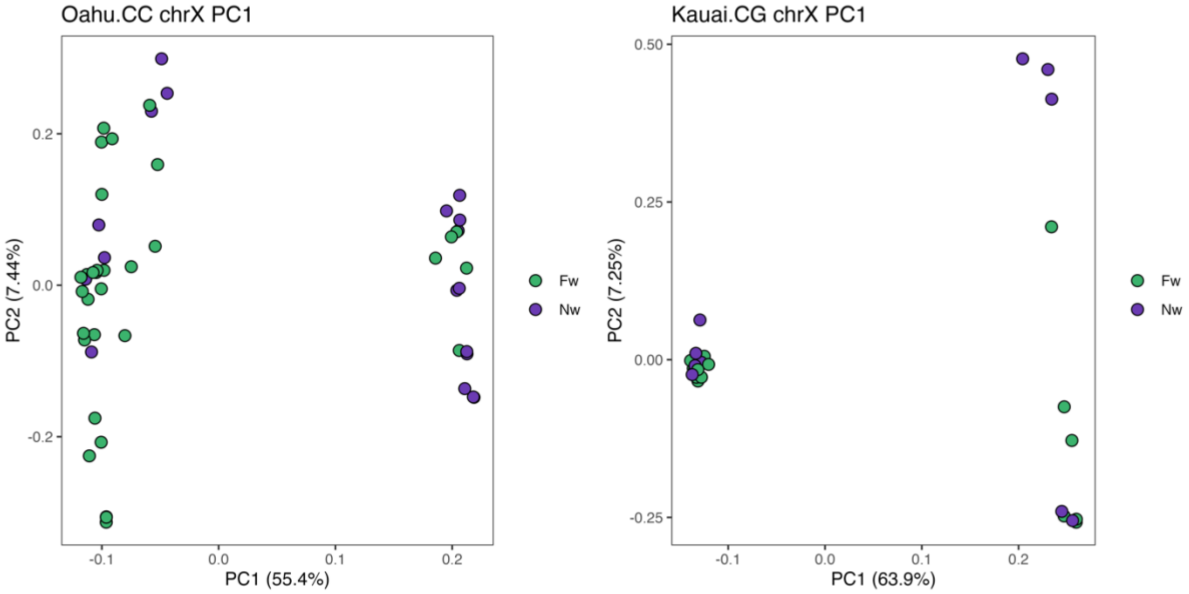

Figure S12. PCAs of variants on Chr1 (the X) using samples from Oahu.CC (N=50) and Kauai.CG (N=30). Samples were clearly separated into two clusters, but cluster identity was not obviously associated with Fw/Nw phenotypes.

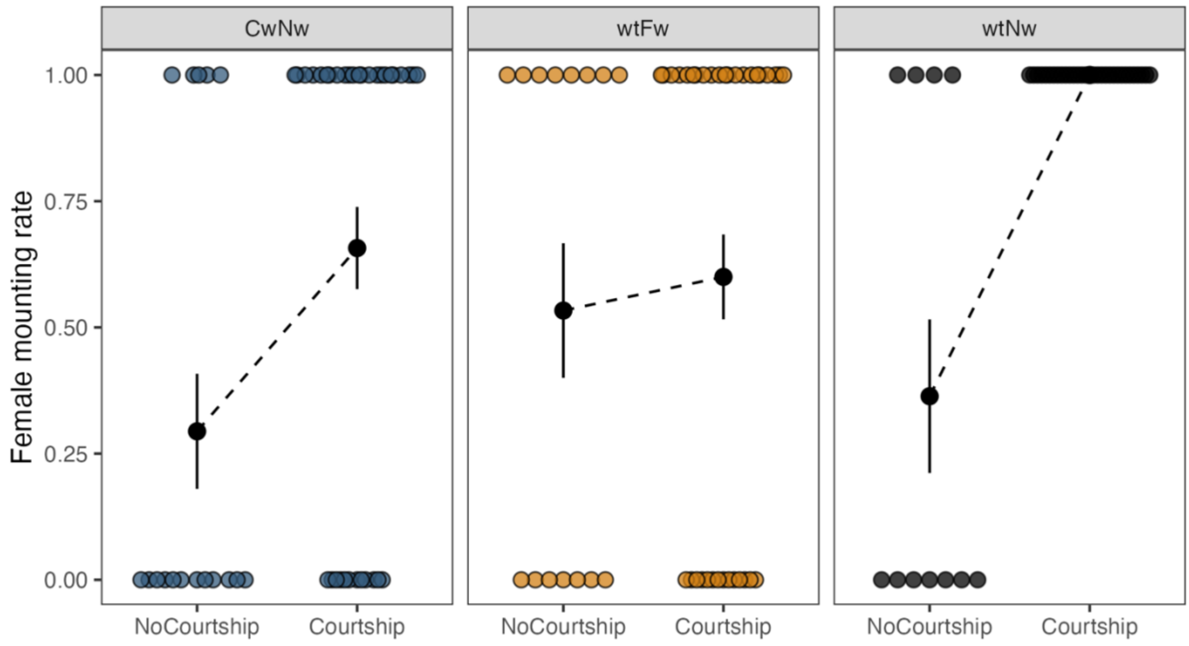

Figure S13. Effects of courtship (measured by attempt to produce courtship song) by males of different wing phenotypes upon rates of female mounting, including repeated measures (cf. Fig. 4A).

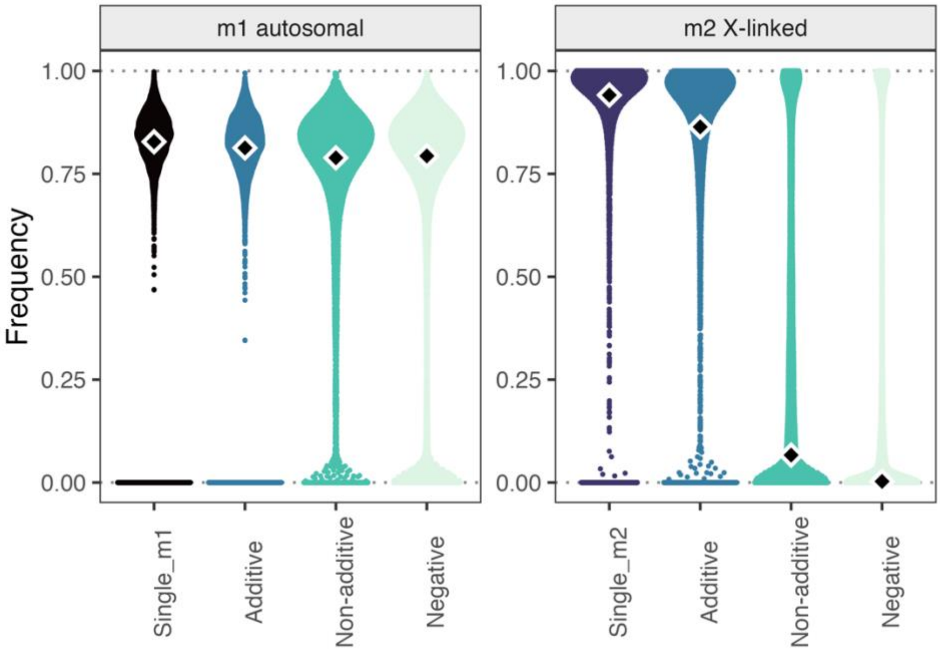

Figure S14. Effects of fitness epistasis on fixation of competing mutations. Frequencies after 100 generations of mutations *m1* (autosomal) and *m2* (X-linked) under scenarios where the mutations combine to have additive, non-additive, or negative effects on fitness of individuals expressing both, versus those expressing either one, and when m1 has a dominance coefficient of 1.0. Note that frequencies of co-occurring mutations are from the same simulations. Diamonds show median frequencies and points are clustered along the X-axis according to their density distribution.

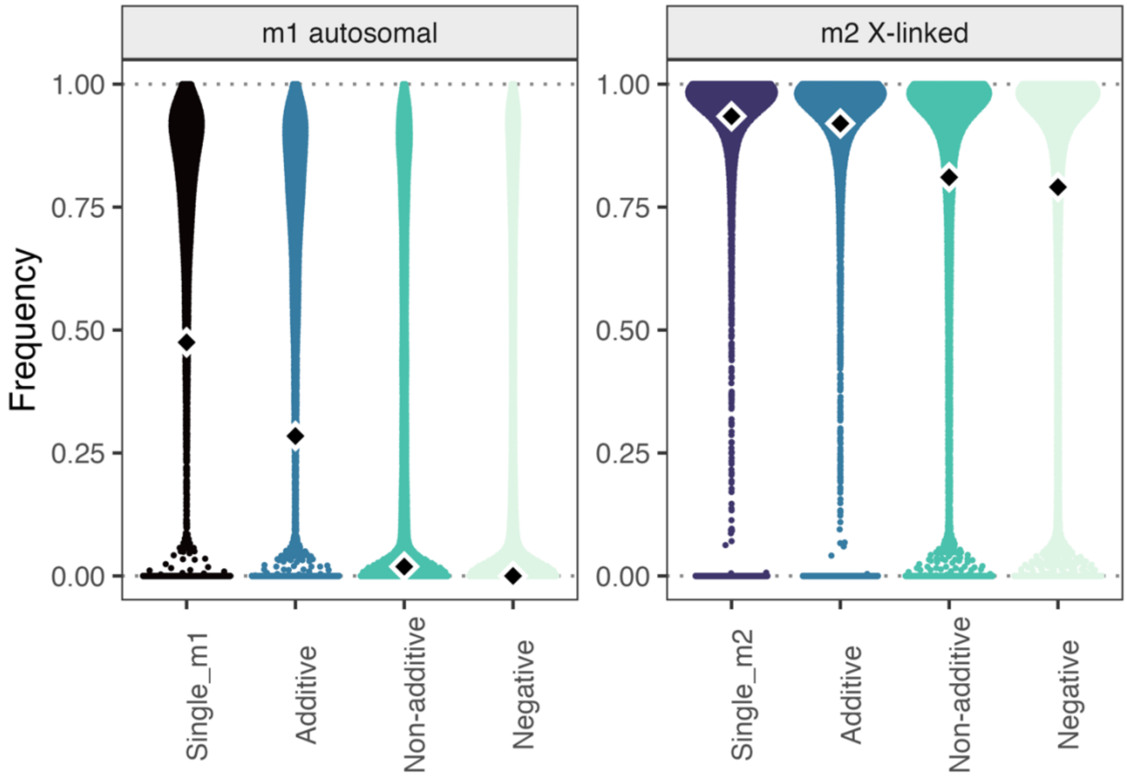

Figure S15. Effects of fitness epistasis on fixation of competing mutations. Frequencies after 100 generations of mutations *m1* (autosomal) and *m2* (X-linked) under scenarios where the mutations combine to have additive, non-additive, or negative effects on fitness of individuals expressing both, versus those expressing either one, and when m1 has a dominance coefficient of 0.5. Diamonds show median frequencies and points are clustered along the X-axis according to their density distribution.

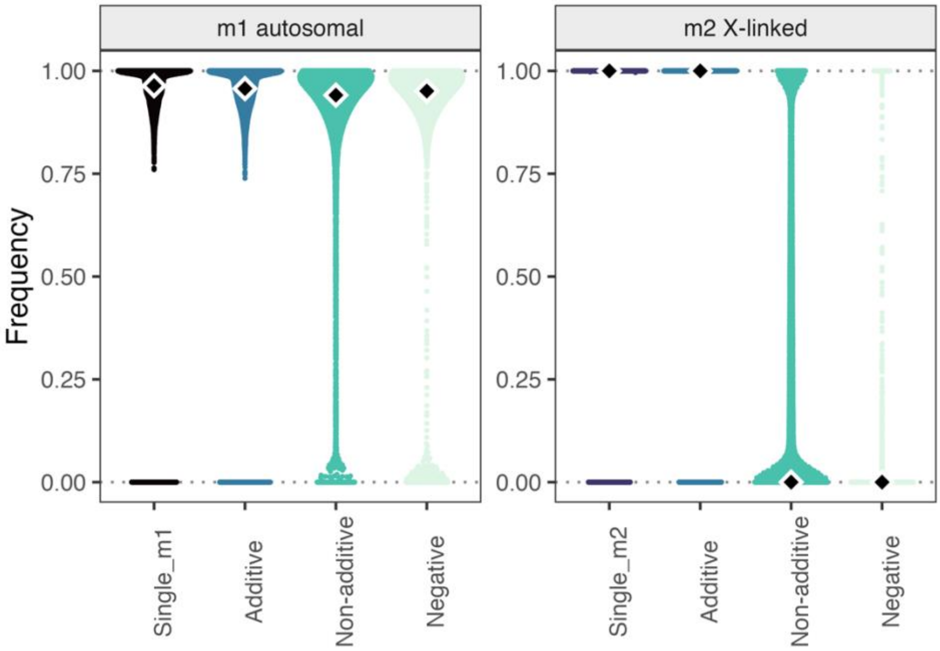

Figure S16. Effects of fitness epistasis on fixation of competing mutations. Frequencies after 250 generations of mutations *m1* (autosomal) and *m2* (X-linked) under scenarios where the mutations combine to have additive, non-additive, or negative effects on fitness of individuals expressing both, versus those expressing either one, and when m1 has a dominance coefficient of 1.0. Diamonds show median frequencies and points are clustered along the X-axis according to their density distribution.

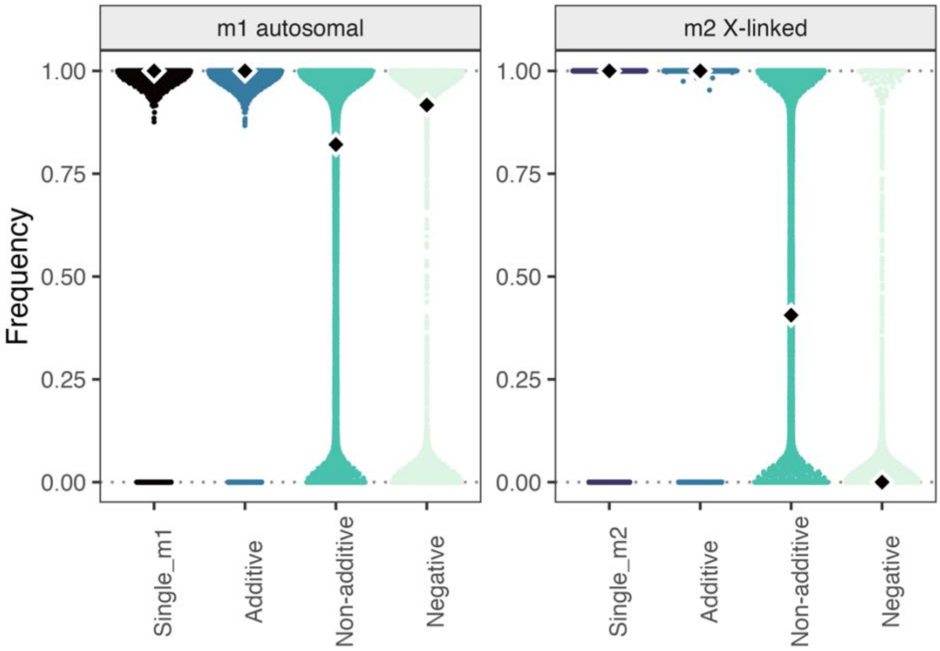

Figure S17. Effects of fitness epistasis on fixation of competing mutations. Frequencies after 250 generations of mutations *m1* (autosomal) and *m2* (X-linked) under scenarios where the mutations combine to have additive, non-additive, or negative effects on fitness of individuals expressing both, versus those expressing either one, and when m1 has a dominance coefficient of 0.75. Diamonds show median frequencies and points are clustered along the X-axis according to their density distribution.

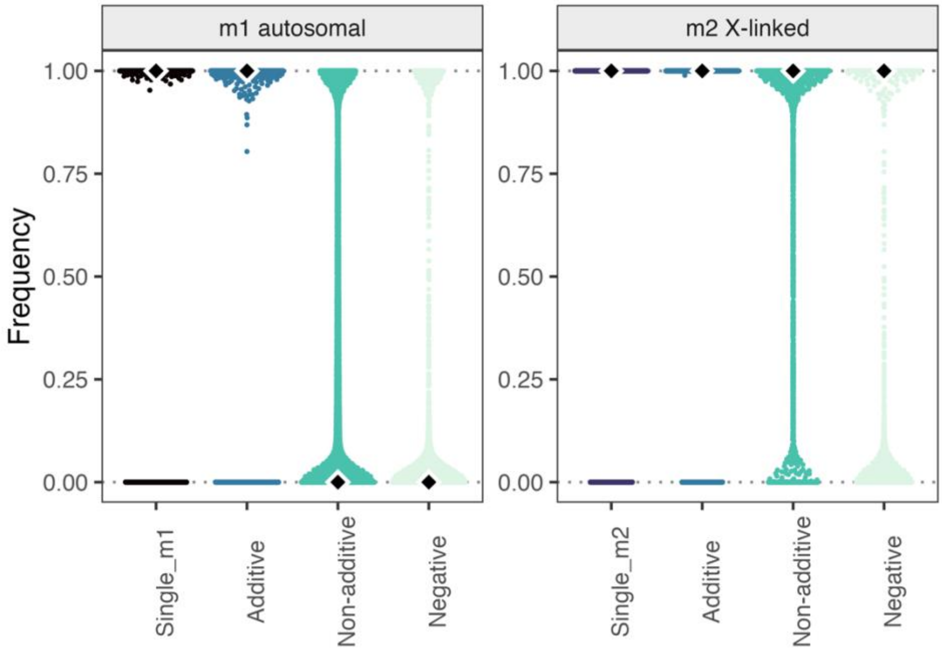

Figure S18. Effects of fitness epistasis on fixation of competing mutations. Frequencies after 250 generations of mutations *m1* (autosomal) and *m2* (X-linked) under scenarios where the mutations combine to have additive, non-additive, or negative effects on fitness of individuals expressing both, versus those expressing either one, and when m1 has a dominance coefficient of 0.5. Diamonds show median frequencies and points are clustered along the X-axis according to their density distribution.

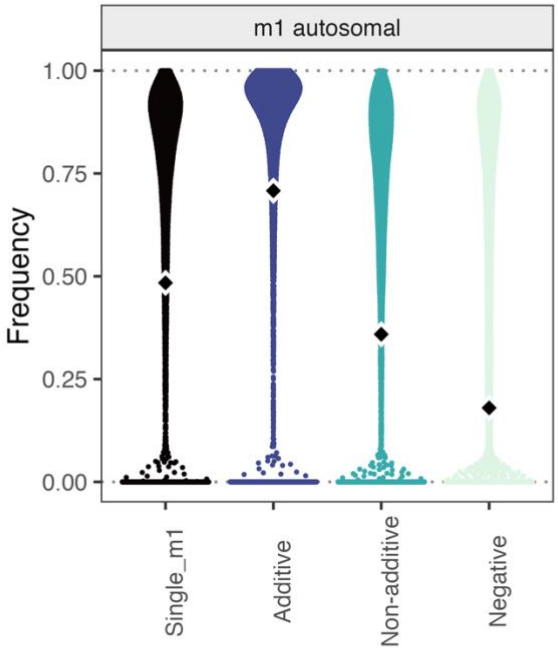

Figure S19. Effects of fitness epistasis on fixation of competing mutations. Frequencies after 100 generations of mutations *m1* and *m2* (both autosomal with dominance coefficients 0.5) under scenarios where the mutations combine to have additive, non-additive, or negative effects on fitness of individuals expressing both, versus those expressing either one. Diamonds show median frequencies and points are clustered along the X-axis according to their density distribution. Since both mutations were autosomal, only m1 is plotted.

Supporting tables

Table S1. Criteria used for scoring wing curliness on a quantitative scale.

Score	Description
0	None; no visible curliness
1	Slight; curliness visible only on close inspection (wing tips or edges slightly upturned)
2	Moderate; readily visible but not striking. Greater than 3/4 of the wing surface retains an ordinary shape.
3	Striking; wing is furled (curls past 180 degrees), or makes little contact with the body (between 1/4 and two-thirds of the wing makes no contact with the body).
4	Extreme; the wing makes almost no contact with the body of the cricket (more than half of the wing makes no contact with the body).

Table S2. Results of a binomial GLMM testing for an association between expression of Cw and expression of Fw among male offspring (*N* = 1,220), including rearing density as a covariate and a nested random effect term representing heritability (1| Father/Mother).

Response		X_1^2	P
Cw expression	Fw expression	0.847	0.357
	Rearing density	7.559	0.006

Table S3. Results of Cox proportional hazards regression for males and females, including wing phenotype and scaled mass index (SMI) as predictors.

	Variable	N	HR	95% CI	p-value
Females	wing_shape	46			
	Cw		2.06	1.67, 2.54	<0.001
	SMI	46	0.99	0.98, 1.0	<0.001
Males	wing_shape	88			
	Cw		1.16	0.52, 2.59	0.7
	wing_veins	88			
	Fw		1.65	0.63, 4.33	0.3
	SMI	88	1.01	0.99, 1.02	0.4

Table S4. Results of a linear mixed model for female mass (across adulthood, with random intercepts for stock box and ID nested within stock box), and linear model for male mass (at 14 days post-adulthood, with a random intercept of stock box).

Response	Predictor	X_1^2	P
Female mass	days	200.232	<0.001
	$days^2$	81.045	<0.001
	wing_shape	5.009	0.025
	days:wing_shape	8.409	0.004
	$days^2$:wing_shape	11.380	<0.001
Male mass	wing_shape	1.225	0.268
	wing_veins	1.247	0.264

Table S5. Results of linear models for female SMI (across adulthood, with random intercepts for stock box and ID nested within stock box), and males (at 14 days post-adulthood, with a random intercept of stock box).

**Response**		**X_1^2**	**P**
Female mass	Intercept	878.224	<0.001
	days	164.907	<0.001
	$days^2$	54.955	<0.001
	wing	0.418	0.518
	days:wing	2.716	0.099
	$days^2$:wing	3.497	0.061
Male mass	Intercept	1346.646	<0.001
	wing_shape	1.219	0.270
	wing_veins	0.324	0.569

Table S6. Description of typical singing-capable and adaptive reduced-song *Teleogryllus oceanicus* wing phenotypes.

<table>
<tr><th></th><th></th><th colspan="2">Wing venation</th></tr>
<tr><th></th><th></th><th>Nw</th><th>Fw</th></tr>
<tr><td rowspan="2">Wing shape</td><td>Wt</td><td>WtNw – the ‘typical’ ancestral male phenotype capable of producing song at ordinary levels due to sexually dimorphic, specialised song-producing structures on the forewing, and the proper engagement of the scraper/file mechanism during stridulation</td><td>WtFw – male unable to produce song at normal amplitude due to the reduction of sound-producing structures on the forewing, including a strongly reduced stridulatory file</td></tr>
<tr><td>Cw</td><td>CwNw – male unable to produce song at normal amplitude due to unusually curled wings precluding proper engagement of the scraper/file mechanism during stridulation</td><td>CwFw – male unable to produce song at normal amplitude due to a combination of the reduction of sound-producing structures on the forewing, and unusually curled wings that preclude proper engagement of the scraper/file mechanism during stridulation</td></tr>
</table>

SUPPORTING REFERENCES

1. K. Bartoń, MuMIn: Multi-model inference. R package version 1.15.6. *Version* **1**, 18 (2016).

2. H. Li, R. Durbin, Fast and accurate short read alignment with Burrows–Wheeler transform. *Bioinformatics* **25**, 1754–1760 (2009).

3. J. Catchen, P. A. Hohenlohe, S. Bassham, A. Amores, W. A. Cresko, Stacks: an analysis tool set for population genomics. *Mol. Ecol.* **22**, 3124–3140 (2013).

4. P. Danecek, *et al.*, The variant call format and VCFtools. *Bioinformatics* **27**, 2156–2158 (2011).

5. S. Purcell, *et al.*, PLINK: A Tool Set for Whole-Genome Association and Population-Based Linkage Analyses. *Am. J. Hum. Genet.* **81**, 559–575 (2007).

6. D. Kim, J. M. Paggi, C. Park, C. Bennett, S. L. Salzberg, Graph-based genome alignment and genotyping with HISAT2 and HISAT-genotype. *Nat. Biotechnol.* **37**, 907–915 (2019).

7. M. Pertea, D. Kim, G. M. Pertea, J. T. Leek, S. L. Salzberg, Transcript-level expression analysis of RNA-seq experiments with HISAT, StringTie and Ballgown. *Nat. Protoc.* **11**, 1650–1667 (2016).

8. M. I. Love, W. Huber, S. Anders, Moderated estimation of fold change and dispersion for RNA-seq data with DESeq2. *Genome Biol.* (2014) https:/doi.org/10.1186/s13059-014-0550-8.

9. H. Mi, *et al.*, Protocol Update for large-scale genome and gene function analysis with the PANTHER classification system (v.14.0). *Nat. Protoc.* **14**, 703–721 (2019).

10. H. Li, A statistical framework for SNP calling, mutation discovery, association mapping and population genetical parameter estimation from sequencing data. *Bioinformatics* **27**, 2987–2993 (2011).

11. J. C. Barrett, B. Fry, J. Maller, M. J. Daly, Haploview: analysis and visualization of LD and haplotype maps. *Bioinformatics* **21**, 263–265 (2005).

12. X. Zhou, M. Stephens, Genome-wide efficient mixed-model analysis for association studies. *Nat. Genet.* **44**, 821–824 (2012).

13. X. Zhang, J. G. Rayner, M. L. Blaxter, N. W. Bailey, Rapid parallel adaptation despite gene flow in silent crickets. *Nat. Commun.* **12**, 50 (2021).

14. S. Pascoal, *et al.*, Rapid convergent evolution in wild crickets. *Curr. Biol.* **24**, 1369–1374 (2014).

15. R Core Team, *R: A language and environment for statistical computing.* (R foundation for Statistical Computing, 2020).

16. D. Bates, Maechler Martin, S. Walker, Package “lme4.” *CRAN Repos.*, 113 (2016).

17. J. Peig, A. J. Green, New perspectives for estimating body condition from mass/length data: The scaled mass index as an alternative method. *Oikos* **118**, 1883–1891 (2009).

18. T. M. Therneau, A Package for Survival Analysis in R (2020).

19. B. C. Haller, P. W. Messer, SLiM 4: Multispecies Eco-Evolutionary Modeling. *Am. Nat.* **201**, E127–E139 (2022).
